# Heparan Sulfate Proteoglycans: Master Regulators of Cellular Signaling, Tissue Development, and Neural Function

**DOI:** 10.1002/jnr.70151

**Published:** 2026-09-01

**Authors:** James Melrose

**Affiliations:** ^1^ Raymond Purves Lab Kolling Institute, Faculty of Medicine and Health, the University of Sydney and Northern Sydney Local Health District, Royal North Shore Hospital Sydney New South Wales Australia; ^2^ Graduate School of Biomedical Engineering, Faculty of Engineering, University of NSW Sydney New South Wales Australia

## Abstract

This narrative review examines the essential roles of heparan sulfate proteoglycans (HSPGs) in brain development, synaptic function, axonal guidance, synapse formation and stabilization, and the establishment of correctly interconnected neural networks. Distinct HSPG profiles help shape precise neural circuits, whereas HSPG dysregulation is associated with impaired neurodevelopmental processes, cognitive decline, and pathological changes in brain tissues. These findings emphasize the importance of HSPGs in normal brain function and in disease‐associated tissue dysfunction. HSPGs are a diverse group of proteins with critical roles in the CNS and PNS, including synaptic function, neurodevelopment, neurodegeneration, cell migration, and regulation of neuroprogenitor stem cell niches in the subventricular ependymal zone and subgranular dentate gyrus. Together, the diverse functions of HSPGs place them as master regulators of tissue form and function in health and disease.

## Significance Statement

Heparan sulfate (HS), the most information dense biomolecule in nature, equips at least 16 HS‐proteoglycans (HSPGs) with a diverse range of biological properties that regulate biodiverse physiological processes. HSPGs are key regulators of brain development and maintenance in maturity, and in specific contexts they may contribute to degenerative changes in brain tissues in diseases such as AD, PD, and dementia. The HS side chains of HSPGs are major functional determinants, underscoring the need to decipher the complex glycosaminoglycan sequences that operate in normal and pathological tissues. The HS interactome is highly complex and, in theory, can generate more than 1,000,000 structurally distinct interactive HS octasaccharide motifs. Although this presents a substantial analytical challenge, significant advances in glycomics are enabling meaningful progress into this difficult analytical area. These advances have supported the development of HS biomimetics, which offer promising opportunities in the regulation of HS‐mediated biological processes, providing insight into the complex roles that glycosaminoglycans perform in situ as components of tissue HSPGs. Three HSPGs in particular (neurexin, pikachurin, and Eyes‐shut) equip the neuron with electrosensory properties responsible for the control of a diverse range of tissue functions.

## Graphical abstract

Ubiquitous cell‐associated, cytoplasmic, synaptic and extracellular matrix HS‐proteoglycans are multifunctional regulators of tissue form and function. HS‐proteoglycans have diverse functions as growth factor co‐receptors in cell proliferation, differentiation, tissue development, mechanical stabilization, synaptogenesis, synaptic plasticity, and neural development important in cognitive learning, problem solving, and memory processes.

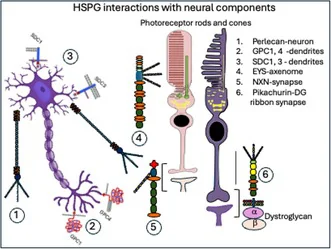

**Abbreviations**
AD: Alzheimers disease
AKT: protein kinase B
ALS: amyotropic lateral sclerosis
AP1: activator protein 1
AT: antithrombin
BBB: blood brain barrier
CNS: central nervous system
c‐Raf: proto‐oncogene serine/threonine‐protein kinase
ECM: extracellular matrix
ERK1/2: extracellular signal‐regulated kinases 1 and 2
EXT: exostosin
EXTL: Nacetylhexosaminyltransferase
FAK: focal adhesion kinase
FGF: fibroblast growth factor
FGFR: fibroblast growth factor receptor
GAG: glycosaminoglycan
GlcA: glucuronic acid
GlcNAc: N‐acetylglucosamine
GlcNAc6S: N‐acetylglucosamine‐6‐sulfate
GlcNS: glucosamine sulfate
GlcNS3S: glucosamine sulfate‐3‐disulfate
GlcNS3S6S: glucosamine sulfate‐3,6‐trisulfate
GlcNS6S: glucosamine sulfate‐6‐dis ulfate
GPC: glypican
HCII: heparin cofactor II
HD: Huntington's disease
HS: heparan sulfate
HS6ST: HS 6‐sulfotransferase
HSPG: heparan sulfate proteoglycan
IdoA: iduronic acid
IdoA2S: iduronic acid‐2‐sulfate
ITGαVβ3 receptor: integrin αVβ3 receptor
JNK: c‐Jun N‐terminal kinase
LG1, 2: laminin G domains 1 and 2
MEK1/2, (MAPK): mitogen‐activated protein kinase
mTOR: mammalian target of rapamycin
NDST: N‐deacetylase/N‐sulfotransferase
NRP‐1: neuropilin‐1
PD: Parkinson's disease
PDK1: phosphoinositide‐dependent protein kinase‐1
PI3: phosphatidylinositol‐3‐kinase/serine/threonine kinase
PKC: protein kinase C
PNS: peripheral nervous system
SARS CoV‐2: severe acute respiratory syndrome coronavirus 2
SDC: syndecan
Shh: sonic hedgehog
SPOCK (testican): SPARC/osteonectin, CWCV and Kazal‐like domain proteoglycan
Src kinase family: Src family non‐receptor tyrosine kinase
TSP: thrombospondin
VEGF: vascular endothelial cell growth factor
VEGFR: vascular endothelial cell growth factor receptor
Wnt: wingless‐related integration site

## 1 Introduction

Heparan sulfate proteoglycans (HSPGs) are diverse (Kirn‐Safran et al. 2009), ubiquitous, cell surface (Hayashida et al. 2022; Kirn‐Safran et al. 2009; Tumova et al. 2000), nuclear (Hayes and Melrose 2021a), intracellular (Boxer and Aoto 2022; Kolset and Tveit 2008; Melrose 2026; Orlandi et al. 2018), and extracellular matrix (ECM) proteoglycans (Greco et al. 2025; Li and Spillmann 2012) (Table 1), that promote cell signaling by acting as growth factor co‐receptors (Woods and Couchman 1992), and are critical to tissue homeostasis, tissue health (Whitelock and Melrose 2011), stem cell regulation (Ravikumar et al. 2020), and the fine tuning of tissue development (Bishop et al. 2007). HSPGs have diverse roles in neurogenesis promoting neural interconnectivity through axonal guidance, synaptogenesis (Yamaguchi 2001), and visual processes (Melrose 2026; Wishart and Lovicu 2021; Wishart and Lovicu 2022; Wishart and Lovicu 2023) (Table 2). Perlecan is an essential component of the neural stem niche and captures FGF‐2 required for the viability of the quiescent slowly recycling stem cell population and for cell signaling in the niche that leads to development of stem cell pluripotency (Kerever et al. 2014; Kerever et al. 2007). HS serves as a molecular mediator, providing instructional cues that regulate cellular activity and gene expression. The evolutionary conservation of HS highlights its importance in cell biology (Yamada et al. 2011).

**TABLE 1:** Extracellular, cell membrane, cytoplasmic granule, photoreceptor, and synaptic HSPGs.

Proteoglycan localization	Proteoglycan	References
ECM	Type XVIII collagen	Marneros and Olsen (2005); Whitelock and Melrose (2011)
ECM	Agrin	Cole and Halfter (1996)
ECM, intracellular, nuclear	Perlecan	Hayes and Melrose (2021a); Hayes and Melrose (2021b)
ECM	SPOCK/testican	Cifuentes‐Dia et al. (2000)
Cell‐surface	Glypican 1–6	Filmus et al. (2008)
Cell‐surface	Syndecan 1–4	Afratis et al. (2017)
Cell‐surface	Betaglycan	Bilandzic and Stenvers (2011)
Cytoplasmic	Serglycin	Kolset and Tveit (2008)
Bipolar neuron ribbon synapse	Pikachurin	Orlandi et al. (2018)
Photoreceptor axoneme	Eyes‐shut	Liu et al. (2020)
Synapses	Neurexins	(Südhof 2017)
Dual substituted HS/CS receptors	Betaglycan	Bilandzic and Stenvers (2011)
	CD47	Gheibihayat et al. (2021)

**TABLE 2:** HS‐proteoglycans regulate diverse cellular and physiological processes aiding in the maintenance of tissue homeostasis and optimal tissue function.

HSPG functional properties	References
Function of HSPGs in basement membranes	Paulsson (1992); Timpl (1993)
HSPGs are integral components of basement membranes (perlecan, agrin, and collagen XVIII), where they form stabilizing networks ensuring tissue integrity and provide an interactive surface for cell attachment and migration. HSPG (perlecan, collagen XVIII, and Agrin) networks with structural proteins stabilize and functionalise the vascular basement membrane and BBB.	
Secretory vesicle HSPGs	Kolset and Tveit (2008)
HSPGs in secretory vesicles such as serglycin form granules that store a range of proteases in an active form and bioactive mediators which are released locally in tissues when these de‐granulate regulating coagulation, immune host defense, wound repair, and inflammation.	
Interaction of cytokines, chemokines, growth factors, and morphogens with HSPGs	Bernfield and Sanderson (1990); Lin (2004)
HSPGs sequester cytokines, chemokines, growth factors, and morphogens and protect these from proteolytic degradation presenting these to and activating their cognate receptors. Formation of growth factor and morphogen gradients drive tissue developmental processes.	
HSPGs as protease and protease inhibitor receptors	Troeberg et al. (2014)
HSPGs can act as protease and protease inhibitor receptors dynamically regulating their spatial distribution and activity in tissue remodeling in repair responses to ECM damage in trauma and disease.	
Interaction of HSPGs with integrins and cell adhesion molecules	Couchman et al. (2015)
Membrane HSPGs cooperate with integrins and other cell adhesion receptors to facilitate cell‐ECM attachment, cell–cell interactions, and cell motility in tissue development.	
HSPG co‐receptors for tyrosine kinase growth factors	Forsten‐Williams et al. (2005)
Membrane HSPGs act as coreceptors for tyrosine kinase‐type growth factor receptors, lowering their activation threshold regulating the duration of cell signaling responses.	
HSPGs as endocytic receptors	(Park et al. 2017)
Membrane HSPGs (glypican, syndecan) can act as endocytic receptors.	
Specialized synaptic HSPGs	Craig and Kang (2007)
Specialized synaptic HSPGs stabilize and provide specificity to neural signaling responses and synaptic plasticity. Ultra rapid vision signaling responses between photoreceptors and bipolar neurons via ribbon synapses facilitate signal transfer to retinal neural networks then to ganglionic neurons through signal transductive transfer to the brain for interpretation.	
HSPG shear flow biosensors	Siegel et al. (2014)
HSPGs can act as shear flow biosensors in blood (perlecan, syndecan, and glypican) sending regulatory cues to endothelial cells and intimal smooth muscle cells regulating gene expression, vasoconstriction, blood volume, and blood pressure. HSPG (perlecan) in the canalicular fluid of bone act as a sensor that regulates osteocyte activity and bone metabolism, flow induced shear stress can also influence differentiation of neuroprogenitor stem cells.	
Cell surface HSPGs facilitate cell‐ECM communication	Guilak et al. (2021)
Cell surface HSPGs or associated with the pericellular matrix facilitate cell‐ECM communication and osmo‐regulation to effect tissue homeostasis and maintain optimal tissue function.	
HSPGs as instructive axonal guidance molecules	Blanchette et al. (2015); Gao, Chen, et al. (2016)
HSPGs provide instructive guidance cues in axonal development and formation of properly interconnected functional neural networks.	
HSPG modules that regulate morphogen activity	de Moraes et al. (2021); Fuerer et al. (2010); Kim et al. (2011); Liu, Wierbowski, and Salic (2022); Rodríguez et al. (2008); Sakane et al. (2012)
Specific core protein domains in HSPGs have been identified that regulate morphogen activities, transport poorly soluble morphogens, and aid in morphogen gradient formation in tissue development.	
HSPGs regulate progenitor stem cells	Kerever et al. (2007)
HSPGs regulate progenitor stem cell function, attainment of pluripotency, and a migratory phenotype in the development of specific stem cell lineages with roles in tissue development and tissue repair capability in wound healing.	
Cell surface HSPGs regulate β‐catenin‐dependent and ‐independent pathways of Wnt signaling.	(Perrimon and Bernfield 2000)
GPC4 bound to Wnt3a and Wnt5a, activate β‐catenin‐dependent and ‐independent pathways, respectively. GPC4 concentrates Wnt3a and Wnt5a close to their specific receptors in different membrane microdomains, thereby regulating distinct Wnt signaling. Specialized cell membrane PG receptors regulate Wnt activity.	
Bioactive matricryptin HSPG fragments	
Many HSPGs contain bioactive cryptic core protein motifs that are released by proteases during tissue turnover or following trauma or disease when protease activity is elevated.	
Synstatin (H‐LPAGEKPEEGEPVLHVEAEPGFTARDKE‐OH), a peptide angiogenesis inhibitor from amino acids 92‐119 of mouse syndecan core protein and the site for αvβ3 and αvβ5 integrin interaction is a critical regulator of these two integrins during angiogenesis and tumourigenesis, SSTN_92‐119_ down regulates ITGαѴβ3 receptor and reduces the activation of angiogenic VEGF and FGF‐2.	Goyal et al. (2011); Goyal et al. (2012); Nyström et al. (2009); Poluzzi et al. (2014)
Perlecan and collagen XVIII contain C‐terminal anti‐angiogenic peptide modules (endorepellin and endostatin). Endorepellin interacts with α2β1 integrin, activates tyrosine phosphatase SHP‐1, dephosphorylates VEGFR2; downregulates VEGFR2 and VEGFA and induces autophagy in endothelial cells. The PI3K/PDK1/Akt/mTOR and PKC/JNK/AP1 pathways are both attenuated. Endostatin interacts with αVβ3 and α5β1 and induces phosphatase dependent activation of caveolin associated Src family kinases and inhibits the FAK/c‐Raf/MEK1/2/p38/ERK1 MAPK pathway.	Poluzzi et al. (2016)
Perlecan domain V and LG3 fragment enhances BBB repair following stroke and displays therapeutic potential in the treatment of vascular dementia. Perlecan domain‐I has been used as a growth factor delivery vehicle in tissue repair. Intact agrin (220 kDa) binds and signals through α‐DG via interactions mediated by its C‐terminal LG1 and LG2 lamG domains and has tissue reparative properties. DG is a cardiomyocyte Agrin receptor, agrin also interacts with neurons. A 44 kDa C‐terminal NT‐1654 Agrin fragment shows tissue reparative properties.	Trout et al. (2021)

Abbreviations: AKT, protein kinase B; AP1, activator protein 1; BBB, blood–brain barrier; c‐Raf, proto‐oncogene serine/threonine‐protein kinase; ERK1/2, extracellular signal‐regulated kinase 1 and 2; FAK, focal adhesion kinase; ITGαѴβ3 receptor, integrin αѴβ3 receptor; JNK, c‐Jun N‐terminal kinase; LG1, 2, Laminin G domains 1 and 2; MEK1/2, also called MAPK, mitogen‐activated protein kinase; mTOR, mammalian target of rapamycin; PDK1, Phosphoinositide‐dependent protein kinase‐1; PI3, phosphatidylinositol‐3‐kinase/serine/threonine kinase; PKC, protein kinase C; VEGF, vascular endothelial cell growth factor; VEGFR, vascular endothelial cell growth factor receptor; Wnt, wingless‐related integration site.

### 1.1 HS Sulfation Has Critical Roles to Play in Neural Development

HS is a GAG which is one of the most information‐rich biopolymers in nature (Whitelock and Iozzo 2005), HS equips at least 16 HS‐proteoglycans (HSPGs) with biodiverse functional properties and these act as effector proteins in a large range of physiological processes (Iozzo and Schaefer 2015).

The sulfation of HS is crucial for the functioning of neural precursor cells during neural development (Karus et al. 2012). Sulfated GAGs including HS play significant roles in the regulation of neural development and regeneration (Kamimura and Maeda 2021). A unique 473HD CS sulfation epitope has been identified in the spinal cord with roles in neuronal maturation and emphasizes the roles of GAG sulfation in neural precursor cell biology. A range of phage antibodies have also been developed to a range of HS sulfation motifs; O‐ and N‐sulfation are important for binding of HS MAbs HS4C3 and HS3G8. Unlike conventionally prepared antibodies, phage display antibodies recognize conformationally defined HS epitopes, rather than sequence alone, and they are also sensitive to cation binding by HS epitopes and may be insightful as to how protein interactions with HS actually occur in situ (Solari et al. 2015). The sulfation pattern of HS is highly conserved between humans and animals; dysfunction in HS sulfation has adverse physiological consequences including neurological dysfunction (Hirano et al. 2025; Xiong et al. 2014). Sulf1 and Sulf2 are cell surface sulfatases, which remove specific 6‐O‐sulfate groups from HSPGs post translationally, resulting in modulation of various HS‐dependent signaling pathways (Kalus et al. 2015). Sulf1 and Sulf2 knockout mice show impairments in brain development and neurite outgrowth deficits illustrating the importance of HS sulfation in neural development. Heparanase also impacts neural development and the pathogenesis of brain tumors, further illustrating the important cell regulatory properties of HS (Xiong et al. 2020).

**FIGURE 1:**
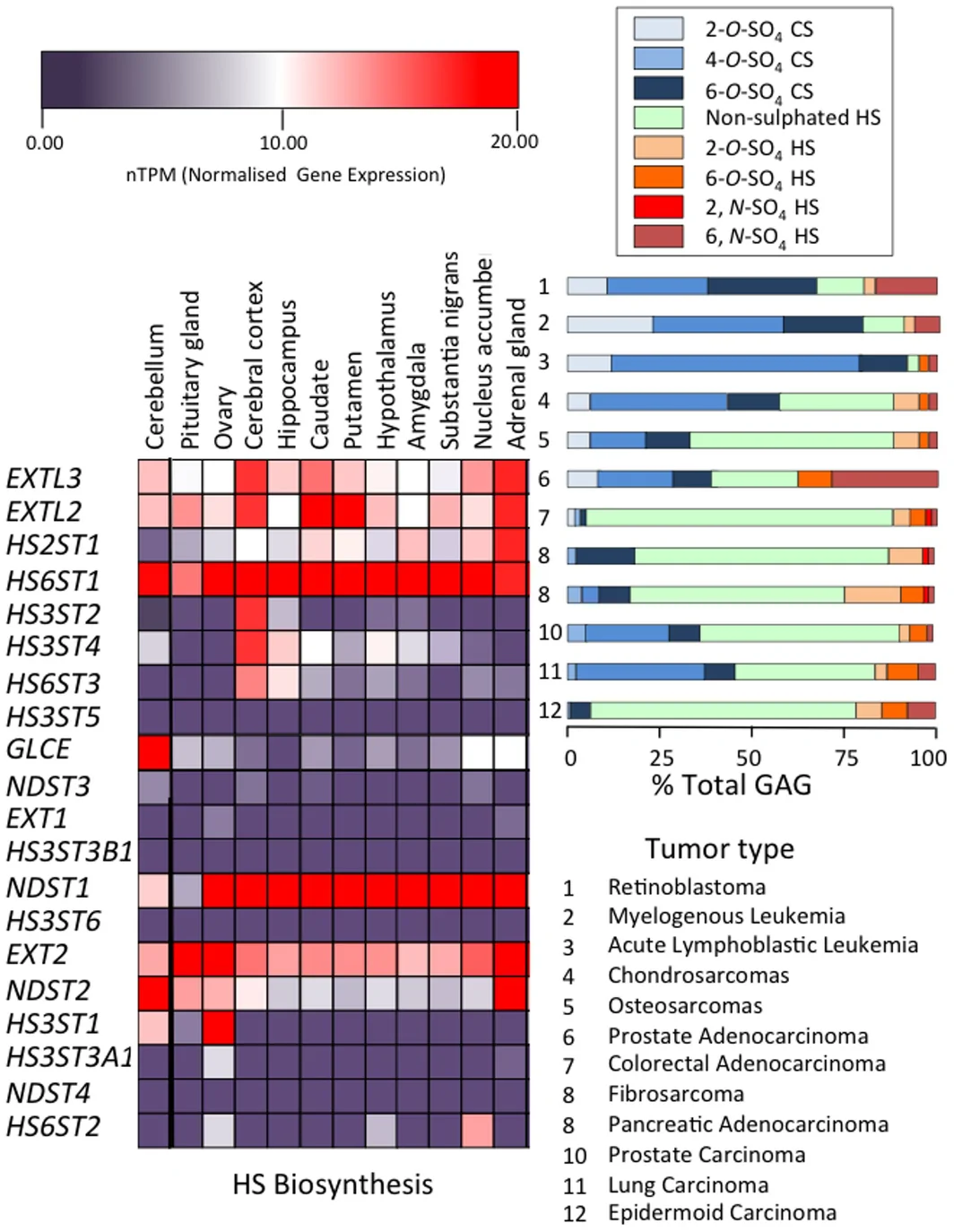
Heatmap depicting the expression of 12 GAG biosynthetic enzymes, demonstrating transcript expression levels for HS in ovary human neural tissues based on RNA sequencing (normalized transcripts per million, nTPM). Data were obtained using the Genotype‐Tissue Expression (GTEx) Program based on The Human Protein Atlas version 21.0 and Ensembl version 103.38. Variation in the sulfation patterns of CS and HS in a number of human cancers. Figure and data modified from Basu et al. (2022). Reproduced under the Creative Commons Attribution CC‐BY 4.0 with permission of The American Physiological Society.

HSPGs are involved in CNS injury responses, modulate growth factor effects, and support neuronal repair (Properzi et al. 2008). Injury‐induced changes in HS sulfation patterns, such as increased 2‐O‐sulfation, are associated with reparative cellular responses in oligodendrocyte precursor cells and astrocytes (Fawcett and Kwok 2022). Variable O‐2, O‐3, and O‐6 sulfation occurs in a diverse range of HS sulfation motifs in a number of tumors in a range of tissues showing the diverse range of motifs that are possible in pathological tissues and are also operative in normal tissue development (Figure 1). The structure of HSPGs is highly variable in normal tissues and is spatio‐temporally distributed in tissues where they regulate subtle variations in tissue development and specialization in specific tissue contexts (Basu et al. 2022; Melrose 2024a). HSPGs fine‐tune tissue composition and the physiological processes they regulate (Bishop et al. 2007).

Glycosylation of proteins and lipids plays a crucial role in numerous biological processes including the regulation of immune and inflammatory responses (Arnold et al. 2007; He et al. 2024). Over 50% of proteins are known to undergo glycosylation and growing evidence suggests that glycosylation plays a pivotal role in cellular differentiation and developmental events, as well as in disease processes. Over 300 mammalian glycosyltransferases have so far been identified (Taniguchi et al. 2014). Sulfation of glycans involves the introduction of a sulfate group onto an oxygen atom, forming a C–O–S bond such as the transfer of a sulfate group to specific hydroxyl groups on glycans, 13 sulfatase genes have been identified in humans (Glatt 2008). The sulfation patterns displayed by glycosaminoglycans are important functional determinants, and where these are present as side chain components on proteoglycans confers important functional properties to these proteins. Table 1 outlines the HSPGs that have been identified and their tissue locations.

## 2 Cell‐Surface HSPGs

### 2.1 The Glypicans

Glypicans (GPCs) regulate key developmental signaling pathways, including those involving Wnt, FGF, VEGF‐A, Hedgehog, BMP, and TGF‐β (Capurro et al. 2014; Capurro et al. 2008; Capurro et al. 2017; Gao et al. 2014; Ho and Kim 2011; Kolluri and Ho 2019; Pan and Ho 2021) and are major cell‐surface proteoglycans of the developing nervous system (Lander et al. 1996). Cerebroglycan (GPC‐2), a 58.6 kDa GPI anchored HSPG with five potential HS attachment sites, significantly influences axonal development but only during early embryonic developmental stages (Ivins et al. 1997). Cerebroglycan, expressed specifically in the nervous system, participates in cell adhesion and regulates axonal guidance and growth during the formation of neural networks. K‐glypican (GPC‐5) is also highly expressed in embryonic brain development (Watanabe et al. 1995) and is developmentally regulated (Saunders et al. 1997). Glypicans are important components of synapse‐organizing protein complexes and serve as ligands for leucine‐rich repeat transmembrane neuronal proteins (LRRTMs), leukocyte common antigen‐related (LAR) family receptor protein tyrosine phosphatases (RPTPs), and G‐protein‐coupled receptor 158 (GPR158), regulating synapse formation and neuronal functional properties (Solari et al. 2015). Recent human genetics studies have revealed that glypicans and HS are associated with autism spectrum disorder, neuroticism, and schizophrenia. Thus, while GPC3 has important roles in normal human development, it also has been identified as a biomarker of glioblastoma and other non CNS tumors (Chan et al. 2013).

### 2.2 The Syndecans

Syndecans 1–4 (SDC1–4) are cell‐surface HSPGs with roles in cell adhesion, intercellular signaling, synaptogenesis, and tissue morphogenesis (Saheki and De Camilli 2012). Neuronal synaptic communication is fundamental to the brain's functional properties (Lee and Sheng 2000). SDC‐2 localizes to synapses, on pre‐ and postsynaptic membranes, where its carboxyl terminus interacts with the PDZ domain of CASK (Calcium/calmodulin dependent serine protein kinase) protein (Hsueh 2006). SDC‐2 accumulates at synapses relatively late during development, where its endocytic roles promote synaptic vesicle recycling, signal transduction, and membrane trafficking (Calabrese et al. 2022; Camblor‐Perujo and Kononenko 2022; Kumari et al. 2010; Saheki and De Camilli 2012; Sorkin and von Zastrow 2009).

## 3 Synaptic HSPGs

### 3.1 The Neurexins

**FIGURE 2:**
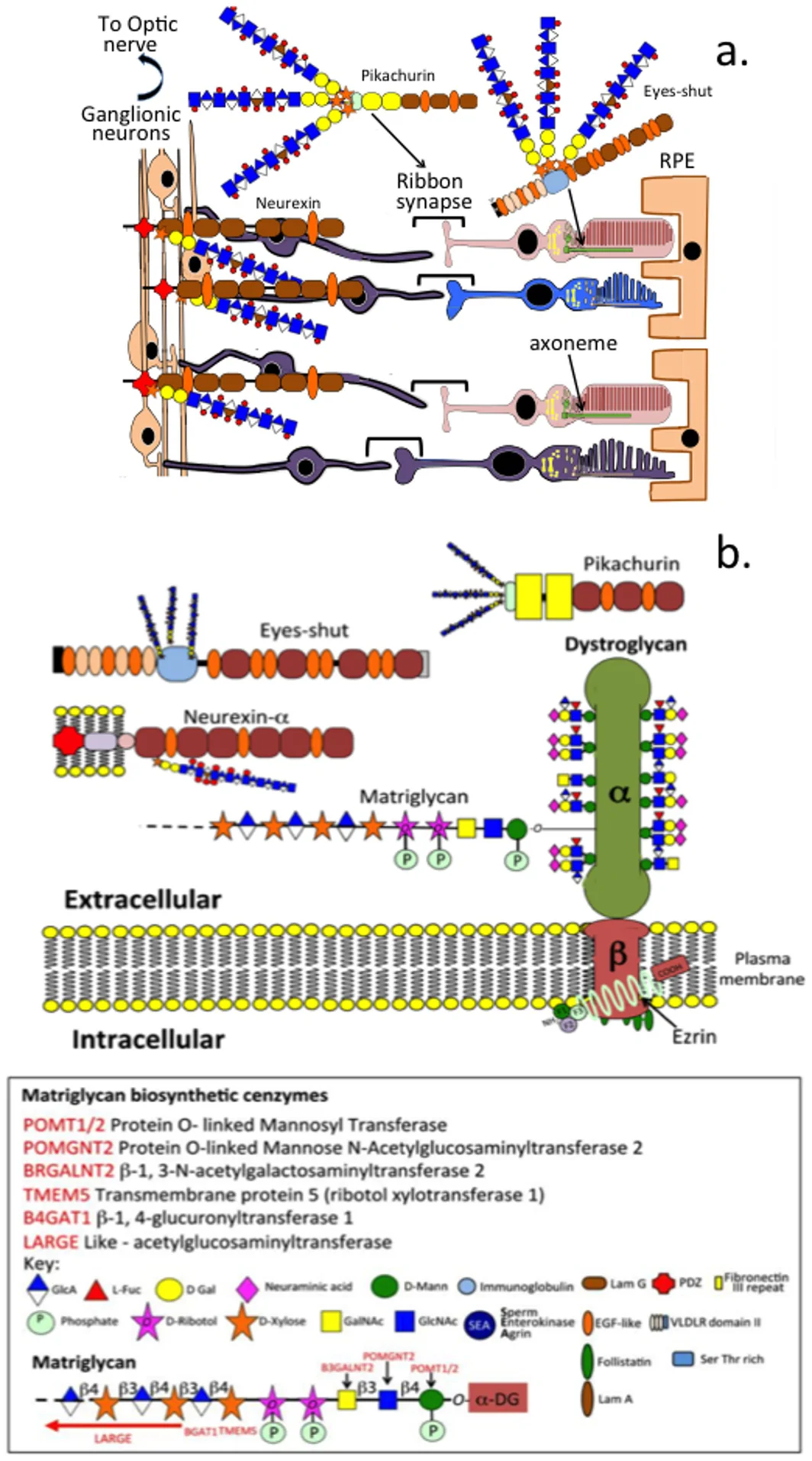
Schematic depiction of synaptic HSPG interactions between ganglionic and bipolar neurons in ribbon synapses in retinal neural networks and interacting with dystroglycan. Structural organization of dystroglycan and glycosyl transferases responsible for the synthesis of matriglycan are also shown. Figure reproduced from Melrose (2024b) using Open Access (Figure Melrose 2024a, 2024b).

The neurexins are specialized synaptic HSPGs (Reissner et al. 2013) with roles in synaptic stabilization mediated by interaction with a large number of synaptic proteins (Figure 2). This also provides specificity to neuronal synaptic interactions and synaptic plasticity. In mammals, NRXN genes produce long α‐neurexin and short β‐neurexin isoforms further diversified by extensive alternative splicing and by HS side chains of variable sequence. Neurexins are key regulators of inhibitory synapses through their interactions with more than 50 extracellular ligands forming a complex, synapse‐specific trans‐synaptic code that shapes inhibitory synaptic properties and local neuronal circuitry (Gomez et al. 2021). This regulates synaptic transmission at inhibitory and excitatory synapses (Uchigashima et al. 2019). Two central studies by Zhang and colleagues and Roppongi and colleagues have defined the complexity of the synaptic interactive proteins that facilitate synaptization with the neurexins (Roppongi et al. 2020; Zhang et al. 2018). Further studies have also emphasized the importance GAGs may play in these neurexin interactions increasing the complex diversity of such interactions (Noborn and Sterky 2021; Suzuki and Yuzaki 2018). Three genes (*NRXN1*‐*3* in humans, *Nrxn1*‐*3* in mice) encode the NRXNs; these undergo alternative splicing at six canonical sites (SS1–SS6), generating thousands of distinct neurexin isoforms, a molecular code providing synaptic specificity and synaptic plasticity (Roppongi et al. 2020; Zhang et al. 2018).

### 3.2 Pikachurin

Pikachurin (EGF‐like, fibronectin type‐III, and laminin G‐like domain‐containing protein, encoded by the EGFLAM gene) is a highly specialized HSPG that interacts with DG and is an essential retinal protein that transmits visual signals via photoreceptor ribbon synapses with bipolar cell dendrites (Melrose 2026). Pikachurin core protein LamG domains interact with α‐DG facilitating an extremely rapid response in ribbon synapse signaling essential in visual processing. Pikachurin dystrophin‐dystroglycan complexes and GPR179 orphan receptor in bipolar neurons facilitate phototransduction of ocular signals. GPR179 complexes with mGluR6 (metabotropic glutamate receptor 6) and TRPM1 (transient receptor potential cation channel subfamily M member 1) facilitate signaling in ON‐bipolar cells (ON‐BC) regulating G protein‐coupled receptor (GPCR) visual inputs from photoreceptors (Koike et al. 2010; Orlandi et al. 2018). Hyperpolarization of photoreceptors in response to light transports glutamate to ON BC mGluR6 initiating a G‐protein signaling cascade regulated by mGluR6 and TRPM1.

### 3.3 Eyes‐Shut

Eyes shut (EYS)‐DG (Melrose 2026) interactions stabilize the inner and outer segments of photoreceptors via the primary cilium axoneme (Yu et al. 2016) through interaction with the matriglycan component of DG (Yu et al. 2018) and is essential for phototransduction (Guo et al. 2026). Cilium structural defects can cause inherited retinal degeneration (Guo et al. 2026; Liu et al. 2024). The primary cilium participates in key signaling pathways required for photoreceptor development and function, making it an important therapeutic target in the prevention of vision loss (Bujakowska et al. 2017; Chen et al. 2021; Ran and Zhou 2020).

## 4 The Large Extracellular HSPGs

### 4.1 Collagen XVIII

**FIGURE 3:**
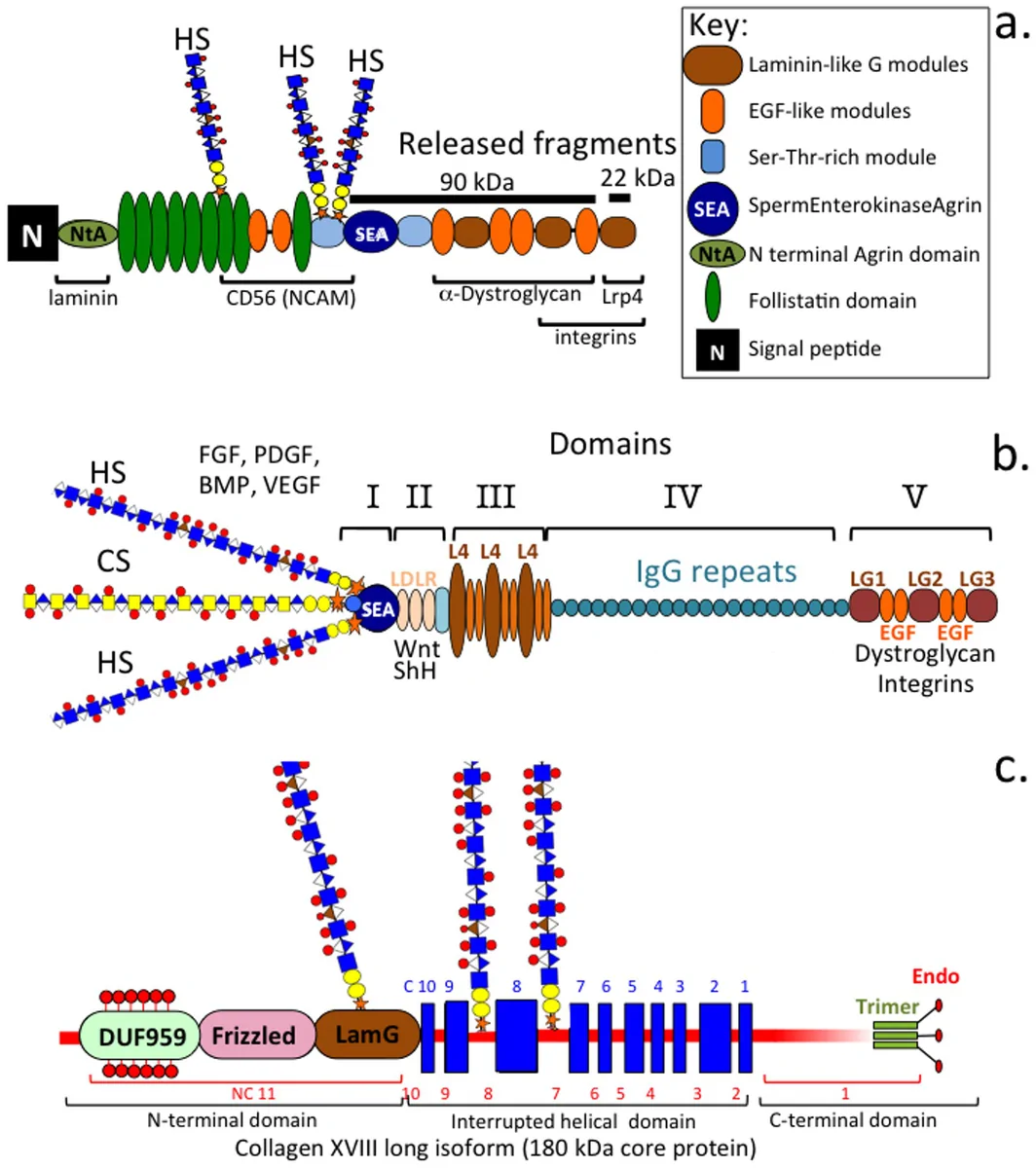
Schematic depiction of the structural organization of agrin (a), perlecan (b), and type XVIII collagen (c). Some interactive ligands or core protein interactive regions are also shown. Figure modified from Melrose (2024b), (figure Melrose 2024a, 2024b).

Collagen XVIII is a basement membrane HSPG expressed as tissue‐specific isoforms that share collagenous and C‐terminal domains but differ in their N‐termini (Saarela et al. 1998). Interrupted collagenous regions provide structural flexibility, while distinct non‐collagenous domains confer diverse biological functions (Figure 3). The long type XVIII isoform contains a frizzled‐like domain with Wnt‐inhibitory activity (Lavergne et al. 2011); the DUF959 domain is of unknown function. A laminin‐G/TSP‐1–like region may interact with DG to stabilize the ECM. Proteolytic cleavage releases endostatin from the C‐terminus; this inhibits angiogenesis in experimental models, although clinical efficacy in trials as an antitumor agent has not been realized. Mutations in *COL18A1* cause Knobloch syndrome, characterized by severe ocular dysfunction due to abnormal basement membrane architecture, demonstrating the importance of collagen XVIII in basement membrane integrity and function (Heljasvaara et al. 2017). Studies in animal models show that collagen XVIII plays key roles in basement membrane stability, cell survival, and stem or progenitor cell regulation. In *Caenorhabditis elegans* , the collagen XVIII orthologue CLE‐1 localizes near dendritic spines of GABAergic neurons and regulates synaptic development and maintenance. Mature collagen XVIII interacts with laminin, perlecan, nidogen, and fibulins to stabilize the basement membrane of the neurovascular unit and maintain blood–brain barrier (BBB) integrity and barrier function (Maro et al. 2015).

### 4.2 Agrin

Agrin clusters acetylcholine receptors at the neuromuscular junction via activation of Muscle‐Specific Kinase (MuSK) (Ngo et al. 2007). In the CNS, agrin is expressed by neurons, glia, endothelial cells, and astrocytes (Kröger and Schröder 2002). Agrin accumulates in the vascular basement membrane during BBB formation and contributes to barrier integrity (Benz and Liebner 2020). Following ischemic stroke, agrin and other basement‐membrane proteoglycans contribute to BBB repair. Agrin promotes dendritic elongation and branching by regulating cytoskeletal organization upregulating microtubule‐associated proteins (Bergstrom et al. 2007; Bose et al. 2000; Mantych and Ferreira 2001). Agrin supports neurite outgrowth and growth cone behavior in CNS neurons and may interact with pathways such as Hippo/YAP, which regulate neural development and inflammation (Cheng et al. 2020).

### 4.3 Perlecan

Perlecan is a large (~467 kDa) HSPG that cross‐links ECM components to provide tissue organization, mechanical stabilization, and cell‐ECM communication (Guilak et al. 2021). Perlecan is expressed early in embryonic development and functions as a mechanosensory molecule that links the extracellular environment to intracellular responses (Wilusz et al. 2012). Perlecan consists of five domains (Melrose 2020). Domain I contains a unique SEA (sea urchin sperm protein, enterokinase, agrin) module containing 3 SGD GAG attachment sites and it also binds numerous ECM proteins and growth factors, acting as a co‐receptor regulating growth factor signaling, cell proliferation, and differentiation (Whitelock et al. 2008). Domains II–IV mediate calcium binding, Wnt signaling, and basement‐membrane stabilization through interactions with laminin, fibronectin, nidogen, agrin, and collagen IV and XVIII. Domain III supports cell attachment; laminin‐like domain III of murine perlecan contains an RGDS sequence that supports integrin‐mediated cell attachment (Chakravarti et al. 1995). Domain IV immunoglobulin (IgG) repeats exhibit homology with neural cell adhesion molecules (NCAMs); mouse perlecan contains 14 IgG repeat modules, human perlecan has 22. Recombinant domain IV binds fibronectin, nidogen‐1, nidogen‐2 and has roles in the integration of perlecan into basement membranes and other ECM structures via protein–protein interactions (Hopf et al. 1999). The C‐terminal domain V of perlecan is considered as a fully functional vascular proteoglycan in its own right (Rnjak‐Kovacina et al. 2017). Domain V promotes angiogenesis; however, specific fragments of domain V are anti‐angiogenic and are of interest as antitumor agents. Domain V is neuroprotective (Trout et al. 2021), enhances BBB repair following stroke and is a promising therapeutic candidate for the treatment of brain ischemia, vascular dementia, brain injury, and neurodegenerative disease (Biose et al. 2022; Bix 2013; Bix et al. 2013).

## 5 Specialized HSPGs

A number of specialized intracellular HSPGs have been identified with instructive roles over cellular processes in the CNS/PNS.

### 5.1 Serglycin

Serglycin (SRGN) is a low‐molecular‐weight intracellular proteoglycan substituted with heparin in mast cells and dermatan sulfate (DS) or chondroitin sulfate (CS) in other cell types (Kolset and Tveit 2008). In mast cells, SRGN binds histamine and proteases including chymase, tryptase, and carboxypeptidase; in neutrophils SRGN binds elastase; in cytotoxic T cells it binds granzyme B; and in endothelial cells, tissue‐type plasminogen activator. SRGN associates with TNF‐α in macrophages and stores inflammatory mediators and proteases within secretory granules, which are released in active form upon degranulation at specific tissue locations, amplifying inflammatory responses, and ECM degradation. Mast cells and basophils predominantly express highly sulfated GAGs (heparin, CS‐D, and CS‐E), whereas natural killer (NK) cells and cytotoxic T lymphocytes express lower sulfation C4S/C6S. Monocytes contain primarily C4S, but upon differentiation into macrophages also express CS‐E (Kolset et al. 1983). SRGN plays key roles in neuroinflammation, and in ischemic stroke.

### 5.2 Betaglycan

Betaglycan (Transforming growth factor beta receptor III, TGFBR3) is a multifunctional 250–280 kDa transmembrane dually modified HS‐CS co‐receptor proteoglycan. It contains binding sites for inhibin, FGF‐2, Wnt, and TGF‐β, reflecting its diverse signaling roles (Bilandzic and Stenvers 2011). Its HS chains bind FGF‐2 but are not required for TGF‐β binding. Wnt signaling is regulated independently of TGF‐β activity: HS inhibits, whereas CS promotes Wnt signaling; *N*‐ and *O*‐linked oligosaccharides modulate ligand binding, growth factor‐mediated vascular and cancer cell migration, and the bioactivity of inhibin A and B (Makanji et al. 2007). Betaglycan ectodomains shed by plasmin and MMPs are antagonists of membrane‐bound betaglycan. Betaglycan localisation with inhibin and activin subunits in the human brain promotes FGF‐2‐mediated neural proliferation and differentiation (Massagué et al. 1992).

### 5.3 The Neuropilins

Neuropilin‐1 and 2 (NRP1, 2) transmembrane receptors substituted with CS or HS but not both|, regulate CNS cell signaling, development, and immune activity. In neurons, endothelial, and immune cells, NRPs bind Class 3 semaphorins acting as VEGF co‐receptors promoting endothelial cell migration (Geretti et al. 2008; Schmidt et al. 2010), and well documented roles in neural development, axonal guidance, and neurovascular interactions, adult neural plasticity and disease (Guo and Vander Kooi 2015; Parker et al. 2012).

### 5.4 CD47

CD47 is a widely expressed transmembrane HS/CS Janus proteoglycan co‐receptor in the brain, and a member of the IgG superfamily that binds to integrins, thrombospondin‐1 (TSP‐1), and signals regulatory protein alpha (SIRP‐α) produced by macrophages and dendritic cells regulating cell adhesion, proliferation, macrophage phagocytosis, neutrophil migration, and T, B, and dendritic cell activation (Oldenborg 2013). Binding of SIRPα to CD47 initiates a signaling cascade that inhibits phagocytosis (Zhang et al. 2015). Phosphorylation of immunoreceptor tyrosine‐based inhibition motifs present in the cytoplasmic tail of SIRPα initiates this response; binding and activation of src homology‐2 (SH2)‐domain containing protein tyrosine phosphatases (SHP‐1 and SHP‐2) blocks phagocytosis by preventing the accumulation of myosin‐IIA at the phagocytic synapse (Gholamin et al. 2017; Li et al. 2021). Binding of TSP‐1 to the HS chains of CD47 inhibits T cell receptor signaling (Kaur et al. 2011) and regulates cellular migration, proliferation, and vascular cell survival in innate and adaptive immune regulation (Barclay and Van den Berg 2014; Murata et al. 2014), inhibition of NO signaling supports blood pressure regulation by limiting eNOS activation and endothelial‐dependent vasorelaxation (Bauer et al. 2010).

### 5.5 Testican/SPOCK Proteoglycans

Testicans (SPOCK1–3, SPARC/osteonectin, CWCV and Kazal‐like domain proteoglycans) are a family of modular HSPGs containing follistatin‐like, a SPARC‐homologous calcium‐binding, and unique C‐terminal domains with HS attachment sites (Iozzo and Schaefer 2015). SPOCK‐1 is localized to post‐synaptic hippocampal neurons, NMJ, and BBB (Bonnet et al. 1996); SPOCK‐2 is enriched in cerebellar and white‐matter (Vannahme et al. 1999); and SPOCK‐3 is widely expressed throughout the CNS (Cifuentes‐Dia et al. 2000). SPOCKs are critical for neural development; mutations or knockout models display microencephaly, impaired CNS development, and behavioral abnormalities.

### 5.6 CD44E

Epican, a HS/CS proteoglycan form of CD44 HA receptor is expressed by epidermal keratinocytes (Kugelman et al. 1992; Milstone et al. 1994); however, keratinocytes share an ectodermal origin with the CNS, and astrocyte–keratinocyte coupled networks may spread inflammatory signals from tissues adjacent to the CNS (Hansson and Skiöldebrand 2015). CD44 is highly expressed by astrocytes, contributing to chronic neuroinflammation and the pathogenesis of ALS, AD, PD, and other neurodegenerative diseases (Al‐Dalahmah et al. 2024; Zhang et al. 2025).

Table 2 provides some examples of the diverse functional roles undertaken by HSPGs in a range of tissues.

## 6 HSPGs Are Master Regulators of Tissue Form and Function

**FIGURE 4:**
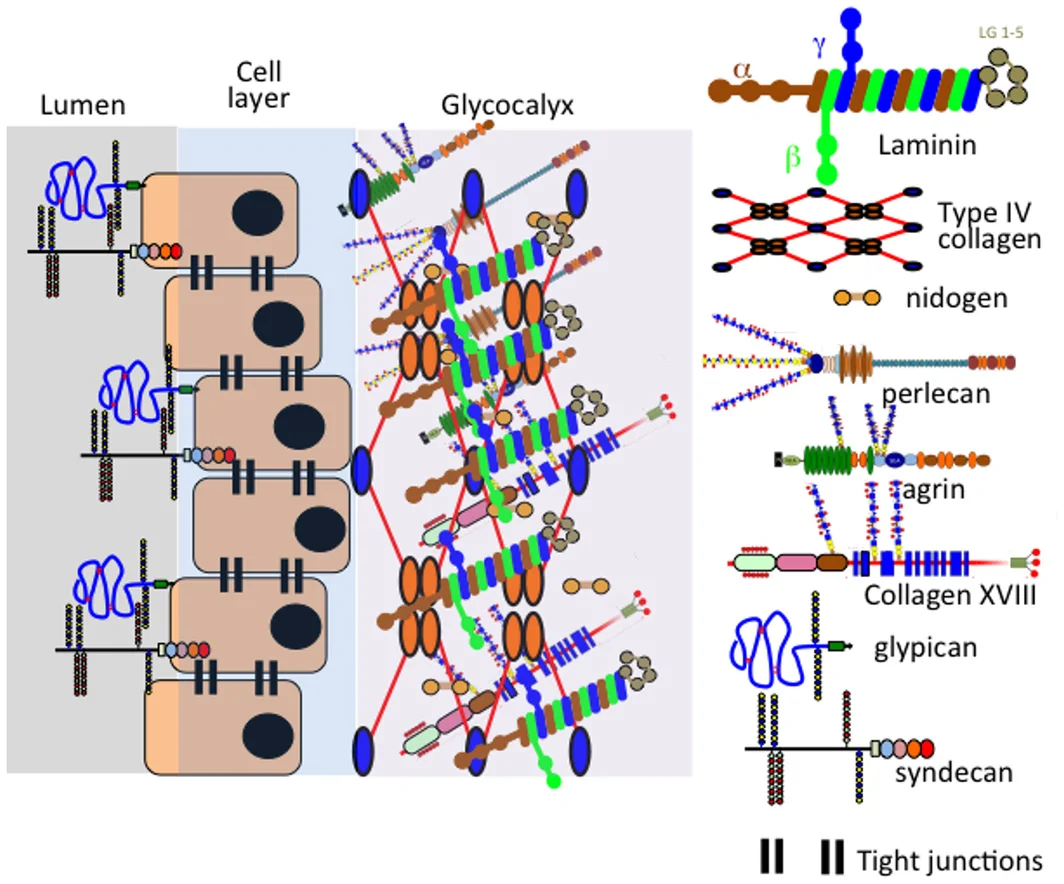
Schematic depiction of the HSPGs that form the glycocalyx in basement membranes of the cerebrovasculature, BBB, and choroid plexus.

**FIGURE 5:**
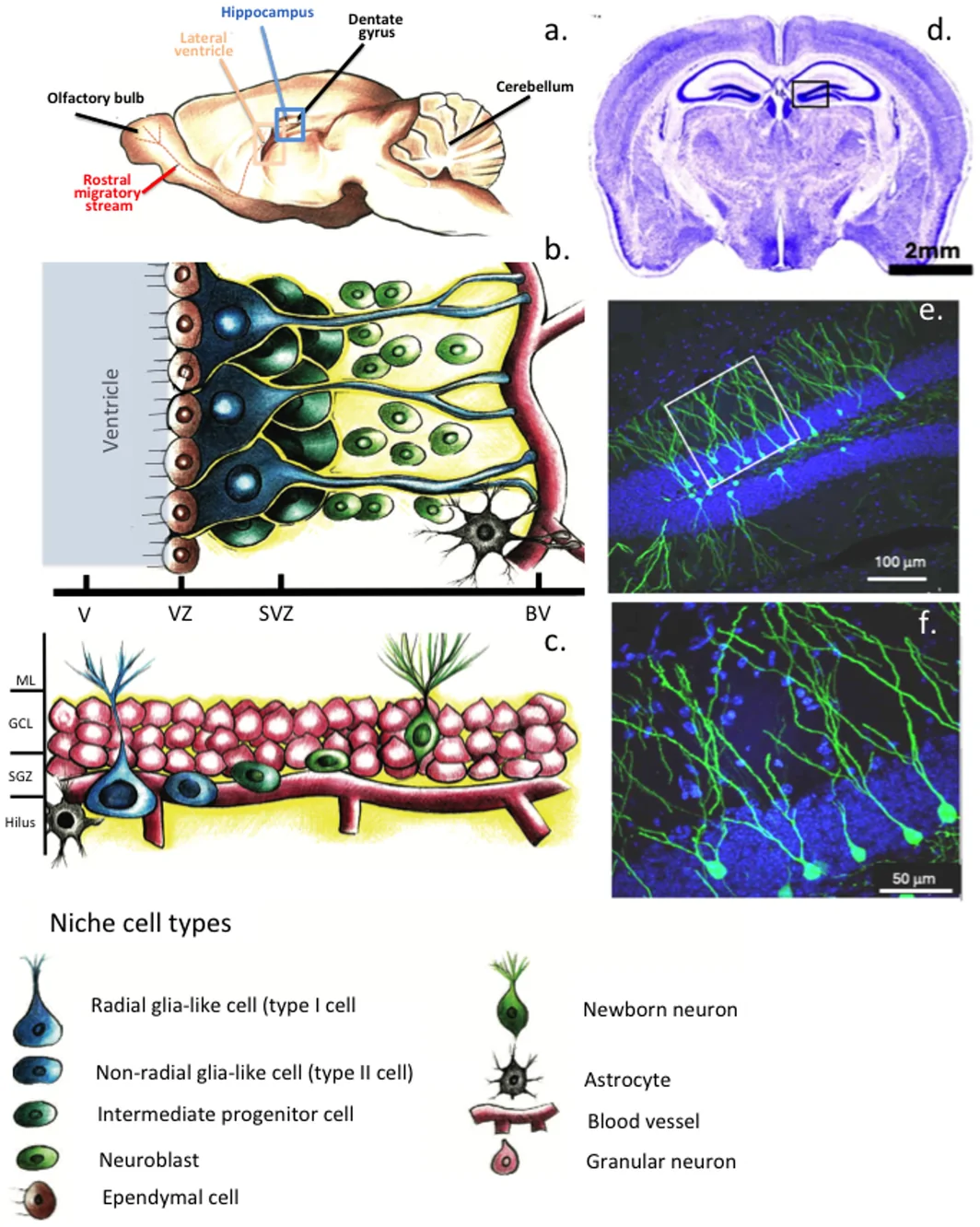
Artistic rendition of the mouse brain and its stem cell locations (a), the sub‐granular dentate gyrus (b), and ependymal sub‐ventricular stem cell niches (c) showing the different cell types and their organization in these niches. Migratory pluripotent stem cells released from these niches migrate to the olfactory bulb via a rostral migratory stream where they are held in reserve till they are required to further tissue development or to initiate a repair response following brain trauma or disease. Figure modified from Regalado‐Santiago et al. (2016), accessed by OpenAccess. BV, blood vessel; SVZ, sub‐ventricular zone; V, ventricle; VZ, ventricle‐ependymal interface; Hilus, indented or depressed region of the dentate gyrus containing astrocytes associated with blood vessels; GCL, granular cell layer; ML, moleculayer layer; SGZ, sub‐granular zone. Emerging pyramidal neurons sprouting from the sub‐granular dentate gyrus are also shown (e, f). Segments (d–f) modified from Vivar and van Praag (2013) Open Access.

HS‐Proteoglycans (Figure 4) are a diverse group of functional proteins (Tables 1 and 2) that play critical roles in the CNS/PNS in synaptic function, neurodevelopment, neurodegeneration, cell migration, development of pluripotent stem cells in sub‐ventricular ependymal and sub‐granular dentate gyrus neuroprogenitor stem cell niches (Figure 5) and the protective barrier functions of the BBB and choroid plexus (CP) (Figure 4). These diverse roles highlight the importance of HSPGs in the maintenance of CNS/PNS function and health.

Advanced glycomics, sequencing, computer assisted molecular docking analyses, and solid phase binding studies have identified specific interactive sequences in HS chains and their binding with bioactive compounds explaining the diverse health promoting properties of HSPGs. The fine structure of HS side chains, including chain length, sulfation patterns, and density, and epimerisation of glucuronic acid to iduronic acid creates a wide range of ligand interactive sites in HS (Ori et al. 2011). Specific HS GAG sequences determine binding affinity and selectivity (Turnbull 2010). Cummings has calculated that a typical interactive HS pentasaccharide composed of repeat disaccharides containing GlcA, IdoA, and IdoA2S at position 1, and GlcNAc, GlcNS, GlcNAc6S, GlcNS6S, GlcNS3S, and GlcNS3S6S at position 2 can yield 2916 possible pentasaccharide combinations (Cummings 2009). The spatiotemporal expression of defined HS structures and HSPG core proteins is tightly regulated during embryonic and skeletal development. HS sulfation is an important functional determinant, and the diversity in structure of the GAG side chains of HSPGs confers cell and organ‐specific functional properties, 3‐*O*, 2‐*O*, and 6‐*O* sulfation motifs in HS equip it with a diverse dynamic recognition system precisely identifying cell regulatory molecules through a “sulfation code” (Turnbull et al. 2001), clearly evident in the sulfation motifs depicted in Figure 1 and reflected in the co‐receptor functions of HSPGs (Hayashida et al. 2022) and the diverse roles they play in the stabilization and functional properties of ocular tissues (Melrose 2026; Wishart and Lovicu 2022), and the CNS/PNS (Fawcett and Kwok 2022). Several HSPGs (perlecan, agrin, and collagen XVIII) form stabilizing functional networks in basement membranes in the BBB, cerebrovascular system, and epithelial cell layer and fenestrated blood vessels of the choroid plexus‐CSF interface, providing protective barrier functions essential to brain health (Figure 4).

### 6.1 Spatio‐Temporally Regulated Tissue HS Cell Regulatory Roles in Health and Disease

**FIGURE 6:**
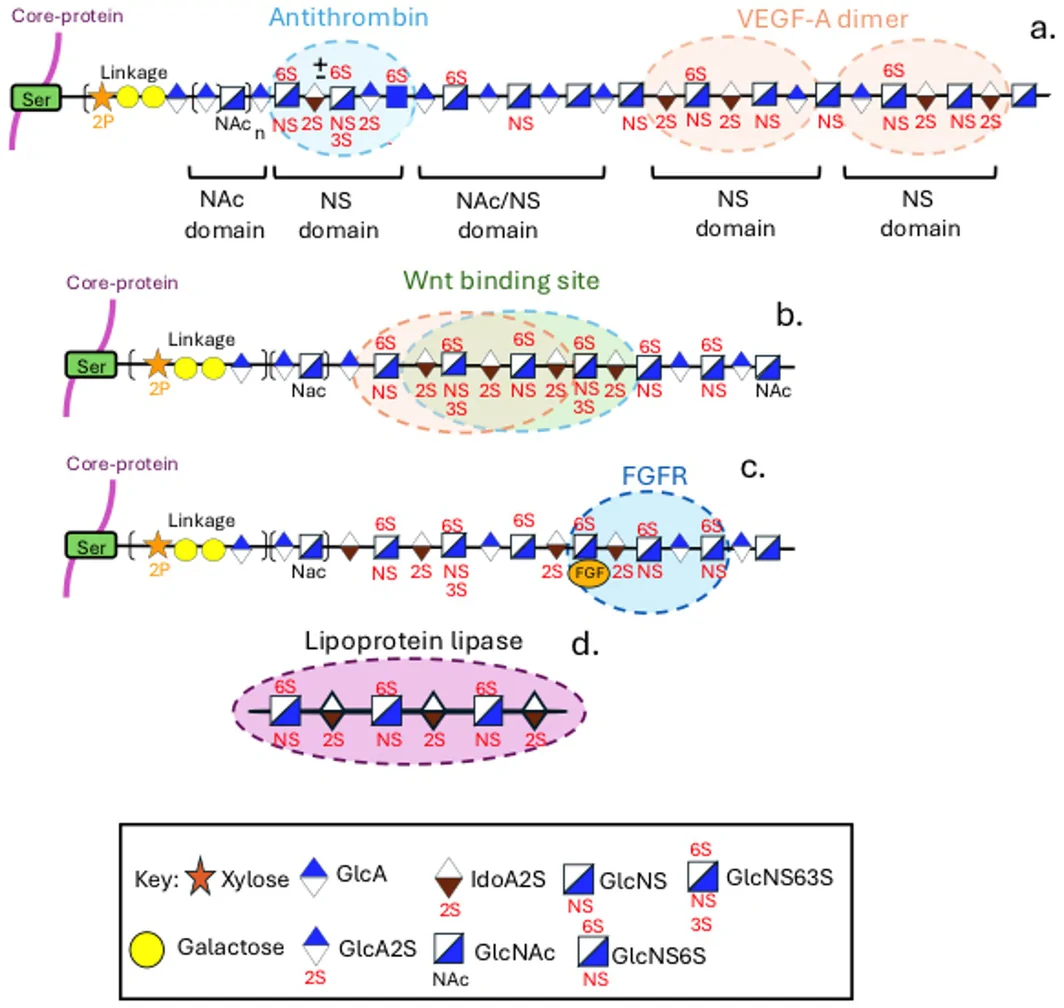
Schematic depictions using SFNG glycan icons of bioactive sequences detected in HS interactive domains for antithrombin and VEGFA dimer (a), the Wnt morphogen 6 saccharide and extended 8 saccharide binding domain (b), FGFR penta saccharide and FGF binding sites (c), lipoprotein lipase 6 saccharide binding site (d). Figure constructed from data presented in (Bishop et al. 2007; Cummings 2009; Gao, Xu, et al. 2016).

Temporal spatial variation in HS structure regulates tissue development, tumorigenesis (Chakraborty et al. 2020), and inflammation (Mousavi et al. 2015). In Figure 1, a heat map depicts the tissue‐specific gene expression of HS biosynthetic enzymes in a number of human brain tissue regions (Basu et al. 2022) and also demonstrates the complexity of HS forms present in a number of tumors. Developmental spatiotemporal expression of HS biosynthetic enzymes in normal tissues also produces HSPGs with GAG side chains of variable structure and roles in tissue development. Glycosylation, the most diverse post‐translational modification of proteins (Wisnovsky and Bertozzi 2022) and sulfation, generates a broad range of ECM instructional cues underpinning cell–cell and cell‐ECM communication and rivals DNA/RNA and protein systems in terms of informational complexity (Wisnovsky and Bertozzi 2022). Glycans however are structurally far more diverse than proteins or nucleic acids and their spatiotemporal variation enables context‐specific regulation of tissue development extending the functional reach of the genetic code, producing an exceptionally broad range of structures with variable abilities to regulate cell proliferation and differentiation and tissue development. Significant improvement in the sequencing of HS and computer‐based binding studies has identified interactive modules in HS with binding properties for antithrombin, VEGFA dimer, Wnt morphogen proteins, FGFR and FGF, and lipoprotein lipase (Figure 6).

### 6.2 HS‐Proteoglycans Provide Axonal Guidance Cues Through Receptor and Neurotrophic Protein Interactions, in Neural Network Formation in the CNS/PNS

Neural precursor cells are stimulated by several families of signaling molecules such as the FGFs, Wnts, and nerve growth factor (NGF) in early phases of brain development (Capossela et al. 2024). HSPGs are key effector molecules in FGF signaling in early brain development. SDC‐1, GPC‐4, and, to a lesser extent, SDC‐4 are expressed in ventricular proliferative zones in early brain development (Ford‐Perriss et al. 2003). Perlecan also has roles in the sequestration of FGF‐2 in ventricular ependymal and dentate gyrus sub‐granular stem cell niches, which ensures survival of the resident slowly recycling quiescent stem cell population and also the development of migratory stem cells of a pluripotent phenotype. These are transported to the olfactory bulb where they reside till required for tissue growth or to elicit a tissue repair response. Blocking HS synthesis inhibits FGF2‐driven neural precursor proliferation, which is rescued by exogenous HS, demonstrating the key roles HSPGs play in neurogenic processes. Stem cells that escape the niche environment receive instructive cues from ECM components such as laminin and perlecan, which not only promotes stem cell proliferation but differentiation to a migratory pluripotent phenotype. These neuroprogenitor cells participate in tissue development and can elicit a repair response when tissues are traumatized or impacted by disease. Oligodendrocyte progenitors originating in the subventricular zone proliferate and also migrate to their final destinations in the brain where they differentiate and interact with axons to form myelinated sheaths on these structures. Oligodendrocyte progenitor development is regulated by growth factors and extracellular cues. HSPGs are key co‐receptors in these processes, promoting cellular proliferation and differentiation. SDC‐3, perlecan, and GPC‐1 direct oligodendrocyte progenitor stem cell development (Winkler et al. 2002).

During embryonic development, neurons must navigate considerable distances to reach their intended final target locations. Syndecans are linked to slit/robo signaling, which aids in axonal migration. During embryonic development, the axons of many neurons migrate along dorsal‐ventral and anterior–posterior axes and may also cross the midline (Kim et al. 2017). Axon migration in the dorsal‐ventral direction is largely controlled by Netrins and the Slits and their Robo receptors. Axonal guidance in the anterior–posterior axis is mainly controlled by Wnts and their Frizzleds/Fz receptors (Killeen and Sybingco 2008; Liang et al. 1997; Smart et al. 2011). HSPGs are essential modulators of longitudinal dopaminergic diencephalospinal guidance by the Netrins (Kastenhuber et al. 2009). Glypican1 and SDC1 and 3 interact with the Robo receptor and stabilize interactions with Slit axonal guidance proteins, GPC3 regulates netrin‐mediated axonal guidance (Blanchette et al. 2015), the SDCs act as cell surface receptors interacting with Slit‐roundabout neural guidance proteins during network formation (Hohenester 2008; Hohenester et al. 2006). Syndecan mutants display misguided phenotypes, inappropriate axonal crossover at the midline, and mispositioned neuronal cell bodies, showing the critical roles these play in normal developmental tissues.

HSPGs and CSPGs direct neural migration and axonal development (Bishop et al. 2007; Díaz‐Balzac et al. 2014) mediated by embryonic BMP (De Cat and David 2001), Wnt (Henríquez and Salinas 2012; Komiya and Habas 2008; Pataki et al. 2015; Varela‐Nallar and Inestrosa 2013), and IHH signaling (Filmus and Capurro 2014; Liu, Wierbowski, and Salic 2022; Perrimon and Bernfield 2000). Wnt proteins form morphogen gradients through interactions between Frizzled and cell‐surface HSPGs, such as the syndecan family (Afratis et al. 2017; Couchman et al. 2015; Kirn‐Safran et al. 2009; Xian et al. 2010). Wnt, SHH, and IHH are hydrophobic, poorly soluble proteins in aqueous environments and rely on transport by perlecan to establish morphogen gradients in tissues (Melrose 2020; Whitelock et al. 2008). This is undertaken by perlecan domain II, an LDLR‐like homology that binds lipids (Melrose 2020). Perlecan also regulates neural cell signaling through interactions with the FGF and the VEGF growth factor families to regulate cerebrovascular development (Iadecola 2017; Lord et al. 2014; Muoio et al. 2014; Whitelock et al. 2008). Neural cells have high metabolic and nutritional demands, and the cerebral vascular system has essential roles to play in supplying their nutrition (Iadecola 2017; Muoio et al. 2014).

**FIGURE 7:**
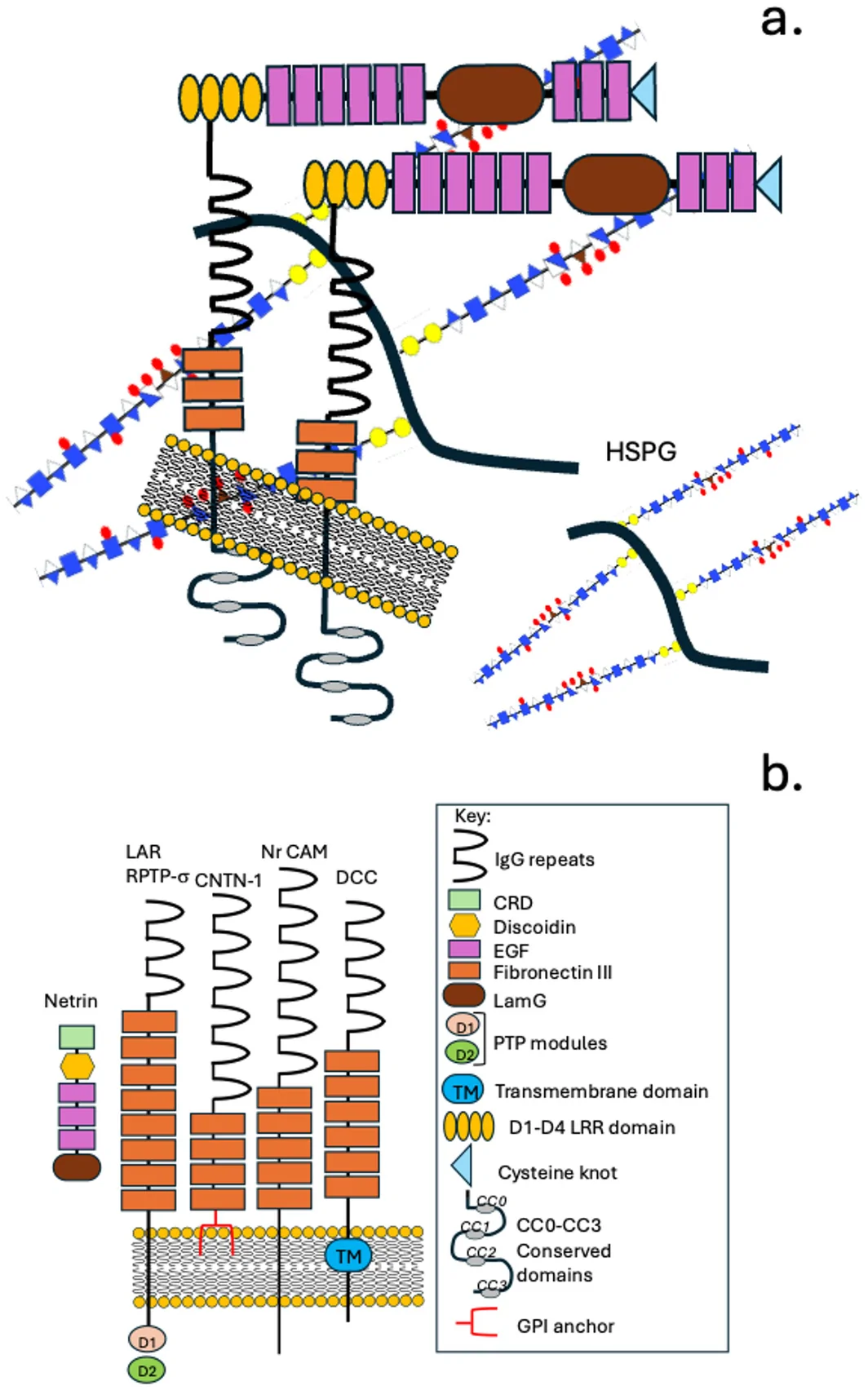
Schematic depiction of the promotion of Slit‐Robo dimerization and activation by HSPGs promotes axonal guidance properties of Slit‐Robo (a). Several other proteins are interactive with HSPGs (b), offering further aspects of axonal guidance in the CNS.

Several members of the receptor protein tyrosine phosphatases (RPTPs) (Fox and Zinn 2005; Maurel et al. 1994), neural cell adhesion molecules (NCAMs) (Akita et al. 2004; Bennett et al. 1997; Kallapur and Akeson 1992), and related contactins (Bouyain and Watkins 2010; Lamprianou et al. 2011; Nikolaienko et al. 2016) act as CS‐ and HSPG receptors at the cell surface. These include the receptor‐linked protein tyrosine phosphatase (LAR), contactin‐1, and neuronal cell adhesion molecule (NrCAM) (Bouyain and Watkins 2010; Lamprianou et al. 2011; Milev et al. 1994; Nikolaienko et al. 2016) (Figure 7).

### 6.3 HSPGs Have Key Roles in the Differentiation of Neural Stem Cells to Neuron, Astrocyte, and Oligodendrocyte Cell Lineages

**FIGURE 8:**
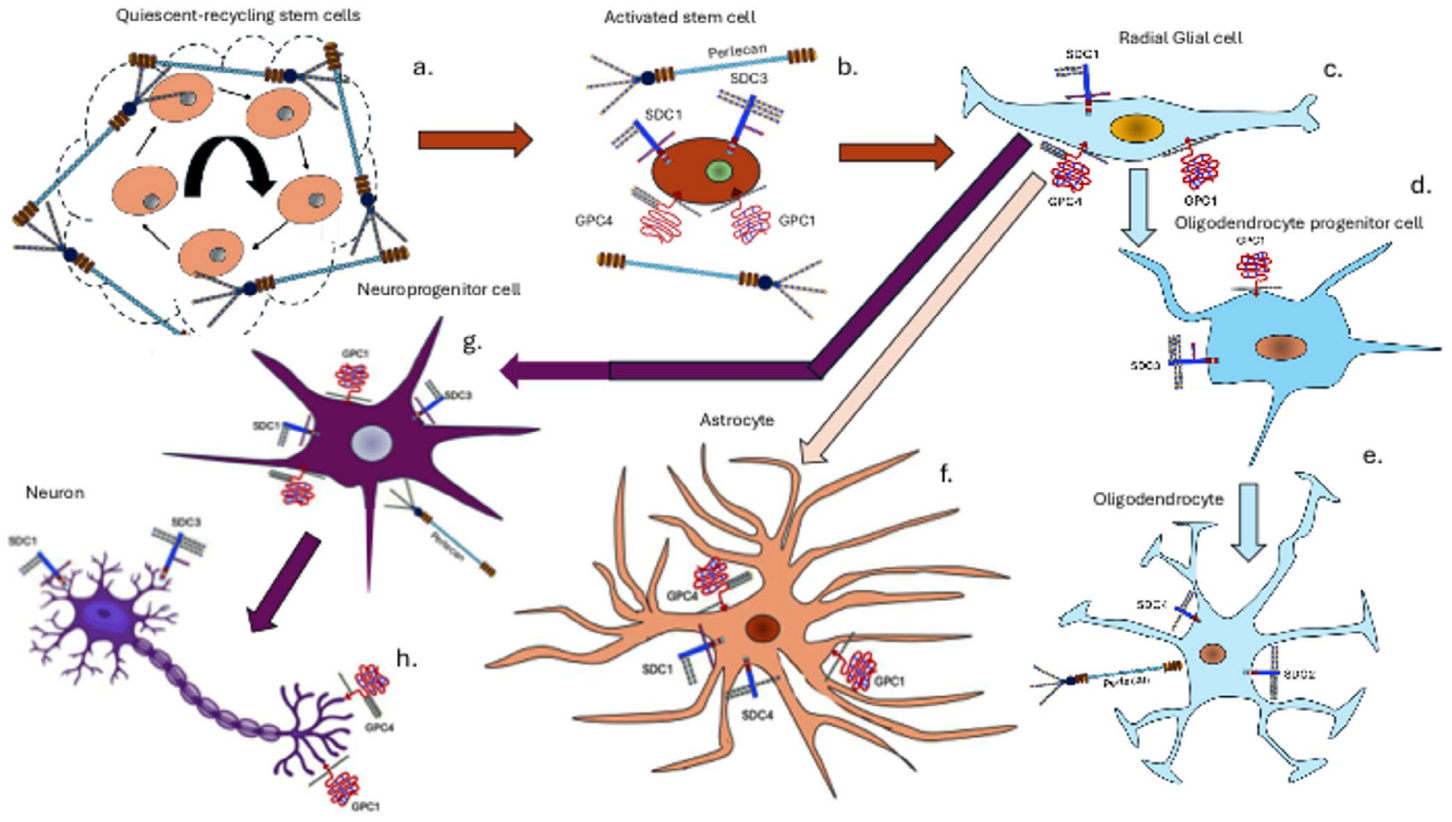
Schematic depiction of the neural stem cell niche, activation of a stem cell and attainment of pluripotency and development of oligodendrocyte, neural, and astrocyte stem cell lineages and the HSPGs associated with these stages of stem cell differentiation.

A number of HSPGs including perlecan and members of the SDC and GPC families have key roles at various stages of the differentiation of neuroprogenitor stem cells during lineage specification into neuron, oligodendrocyte, and astrocytic cell types (O'Connor et al. 2025; Ravikumar et al. 2020; Yu et al. 2017) (Figure 8). Such manipulations may be useful in the preparation of iPSC astrocyte progenitor cells for neural repair applications (O'Connor et al. 2025; Ottoboni et al. 2020; Smith et al. 2022). HSPGs control the bioavailability of a range of growth factors and morphogens during embryogenic development which direct tissue growth (Melrose 2020).

Perlecan has key roles in the maintenance of the quiescently recycling stem cell pool in the stem cell niche through sequestration of FGF‐2 (Kerever et al. 2007; Whitelock and Iozzo 2005). Stem cells that receive signals from the ECM environment of the niche drive them to a pluripotent migratory cell phenotype. Laminin‐1, fibronectin, and collagen‐IV promote the extension of fine cellular processes in iPSC‐derived astrocyte progenitor and neuronal progenitor cells (Whitelock et al. 2008).

Activated neuroepithelial/neural stem cells express SDC1, SDC4, GPC1, GPC4, and perlecan, which mediate cell proliferation and maintain the pluripotent state via FGF and Wnt signaling. Perlecan sequesters FGF‐2 and promotes diverse cell signaling events (Matsuo and Kimura‐Yoshida 2014). Wnt and Hedgehog (Hh) are poorly soluble morphogens; perlecan domain II binds and transports these, establishing morphogen gradients during embryonic development (Kerever et al. 2014).

Radial glial cells have the potential to differentiate into neurons, astrocytes, and oligodendrocytes (Yu et al. 2017). These express SDC1, GPC1, and GPC4; development of a neuronal progenitor lineage results in expression of GPC1, GPC4, SDC2 on dendritic spines and dendritic maturation (Figure 8). SDC3 provides axonal guidance along with GPC1 during synapse formation. Synaptic GPC4 is also observed in mature neurons. With astrocyte lineage specification, SDC1 expression diminishes and SDC4 and GPC6 expression become detectable while GPC1 and GPC4 expression are retained during astrocyte maturation. Perlecan has an inhibitory effect on astrocyte proliferation through domain V (Yu et al. 2017). With oligodendrocyte lineage specification, precursor cells retain SDC1 and GPC1 expression, promoting cell proliferation, but this inhibits differentiation; SDC3 and perlecan expression are also up‐regulated at this stage. In mature oligodendrocytes, SDC2, SDC4, and perlecan are the major HSPGs.

### 6.4 The Choroid Plexus

**FIGURE 9:**
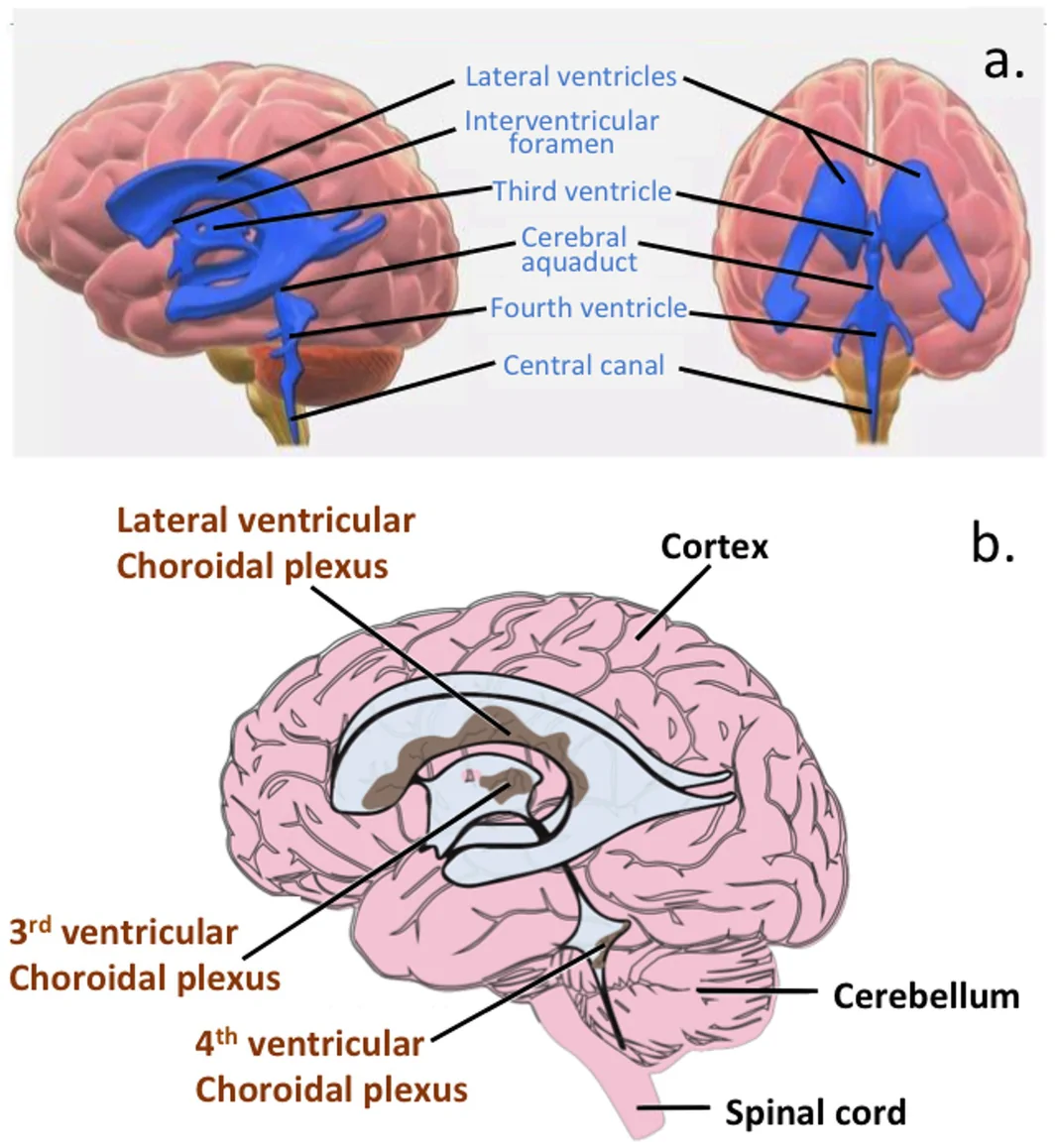
Diagram of the ventricles of the brain (a) and the choroid plexuses within them (b). Adapted from Human Physiology: An Integrated Approach by Dee Silverthorn, 8th edition (2019).

**FIGURE 10:**
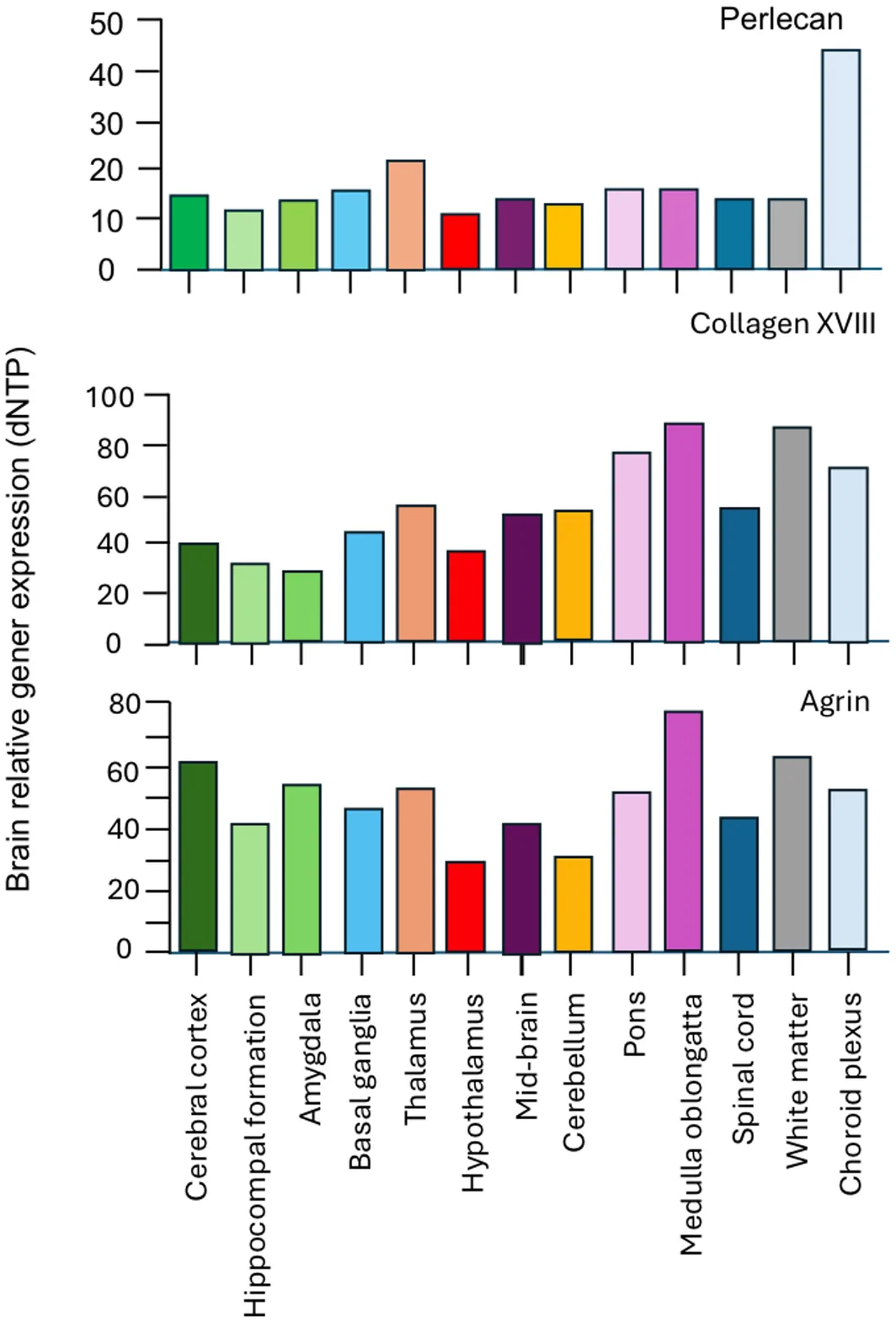
Relative gene expression of HSPGs in different regions of the brain including the choroid plexus. Data from the Human Protein Atlas (Digre and Lindskog 2021; Pontén et al. 2008). The Human Protein Atlas (Human Protein Atlas proteinatlas.org) is licensed under a Creative Commons Attribution 4.0 International License. Data shown is available from https://www.proteinatlas.org/ENSG00000142798‐HSPG2/brain.

Two major protective CNS barriers regulate the movement of molecules between blood, brain and cerebrospinal fluid: the BBB defined by the cerebral micro‐vasculature, and the blood‐CSF barrier of the Choroid Plexus (CP) (Sathyanesan et al. 2012) (Figure 9). The CP of the ventricle system is stabilized like the BBB by perlecan‐collagen XVIII‐Agrin networks and these also support barrier functions (Handler et al. 1997; Roberts et al. 2012; Wolburg and Paulus 2010) and delivery of growth factors important for brain cell nutrition. CSF production by the CP has drawn increasing attention in AD research. Markedly decreased CSF production and turnover occurs in moderate‐to‐severe AD leading to impaired clearance of toxic metabolites, neuroinflammation, and neuronal death. Amyloid β oligomers disrupt blood‐CSF barrier integrity through induction of MMPs (Brkic et al. 2015) leading to neuroinflammation in AD (Balusu et al. 2016). The CP has indispensable functions in the development, maintenance, and functioning of the brain and represents a “smart gate” with protective properties controlling the internal environment of the brain (Brkic et al. 2015; Marques et al. 2017) releasing CSF through ion channels and recruiting immune cells protecting against infection and tissue damage in neuroinflammation (Xu et al. 2024). Growth factors and peptides released by CP epithelial cells support cerebrovascular repair in neurological disease and following stroke (Kaur et al. 2016; Liu, Zhang, et al. 2022). Dysfunction of the CP epithelium results in pathological changes in hydrocephalus, multiple sclerosis, and AD (Balusu et al. 2016; Brkic et al. 2015). Accelerated CP atrophy has been reported in stroke, multiple sclerosis, schizophrenia, and other CNS diseases. Data from the human atlas project (Human Protein Atlas proteinatlas.org) show perlecan, collagen XVIII, and agrin are expressed in the CP and these presumably have structural and functional roles similar to in basement membranes and BBB (Xu et al. 2018) (Figure 10).

## 7 Roles for HSPGs in CNS/PNS Degenerative Disorders

Glycosyltransferases and sulfotransferases are expressed in response to traumatic injury in the CNS/PNS producing HSPGs with variable HS sequences and sulfation patterns. Astrocytes treated with TGF‐alpha or TGF‐beta to mimic an injury response upregulated SDC‐1 and HS2ST activity correlating with an increase in 2‐O‐sulfation of HS and increased staining with the AO4B08 anti‐HS antibody recognizing high HS sulfation (Properzi et al. 2008). Brain cells also upregulate HSPG production in response to trauma which participate in a brain tissue repair response and these modulate growth factor activity, localize neurotrophic molecules, and axon growth (Properzi et al. 2008). After injury, HSPG levels, mRNA for HS2ST, staining with sulfation‐specific antibodies were evident in the rat brain consistent with an increase in 2‐O‐sulfated HS, SDC‐1 was also upregulated in cultured glia and astrocytes. SDCs play key roles in neurodegenerative processes by mediating the internalization of misfolded proteins implicated in neurodegenerative diseases (Hudák and Letoha 2025) contributing to the seeding, aggregation, and toxic spread of protein aggregates. SDC‐3 regulates the uptake and spread of misfolded proteins, fostering neurotoxic aggregates that impair neurons and cause cognitive decline (Song et al. 2014). This is evident in AD, affecting millions globally and is a leading cause of dementia (Kamatham et al. 2024; Letoha et al. 2019). Key features of AD include extracellular accumulation of amyloid‐beta (Aβ) plaques and intracellular formation of tau neurofibrillary tangles, disrupting neuronal function and triggering neuroinflammation (Jellinger 2010; Sadigh‐Eteghad et al. 2015). PD, HD, and ALS also involve the accumulation of misfolded proteins like α‐synuclein and polyglutamine proteins (Chen et al. 2002; Hudák et al. 2019; Ross and Poirierm 2004) that form neurotoxic aggregates contributing to neuronal dysfunction and cell death (Soto and Estrada 2008; Westermark and Westermark 2010).

### 7.1 Neurexins and Neurodegeneration

A clear link has been established between NRXNs and neuropsychiatric disorders, such as autism spectrum disorder and schizophrenia, and changes in NRXN expression are also found in AD and PD (Cuttler et al. 2021). Contactin‐associated protein‐like 4 (CNTNAP4), a member of the NRXN superfamily, has critical functions in neurological development and synaptic function. Loss of CNTNAP4 in interneurons leads to autism, schizophrenia, and epilepsy. CNTNAP4 knockdown in substantia nigra induces PD‐like increases in α‐synuclein expression (Zhang et al. 2020).

### 7.2 The Syndecans and AD

The SDCs display increased expression levels in AD, where they trigger the aggregation and modulation of cellular uptake of Aβ. Some Apolipoprotein E (ApoE) increase the risk for AD, while ApoE2 decreases it. Neuronal SDC3 increases the cellular uptake of ApoEs; however, ApoE2 increases the internalization of monomeric Aβ, preventing its extracellular aggregation, while ApoE4 decreased it, promoting the extraneural buildup of Aβ plaques (Hudák et al. 2021; Hudák et al. 2022).

HSPGs can promote Aβ and tau fibril formation in AD. SDC1, GPC3, and CD44v3 show low expression levels, while SDC3 and GPC1 are highest, SDC4 is overexpressed in tau and Aβ pathology in AD, some HSPGs are thus potential inducers of AD (Lorente‐Gea et al. 2020). Intraneuronal accumulation of Aβ(1‐42) is one of the earliest signs in AD and cell surface HSPGs promote its cellular uptake. The neuron specific SDC‐3 isoform optimally increases cellular uptake of Aβ1‐42 (van Horssen et al. 2003). Cerebrovascular deposition of Aβ occurs in AD and hereditary cerebral hemorrhage with amyloidosis and cerebral amyloid angiopathy. GPC1 is abundantly expressed in cerebral amyloid angiopathy in AD colocalized with agrin. SDC‐2 is also frequently found in vascular Aβ in AD. SDCs, agrin, GPC1 are associated with senile plaques, suggestive of roles in their formation (Regev et al. 2017; van Horssen et al. 2001; van Horssen et al. 2003).

### 7.3 Roles for the Glypicans in AD

Tau protein aggregates are a major driver of neurodegeneration and behavioral impairments in tauopathies, including AD. Apolipoprotein E4 (*APOE4*) is the highest genetic risk factor for late‐onset AD and exacerbates tau hyperphosphorylation in mouse models. APOE4‐mediated surface trafficking of APOE receptor low‐density lipoprotein receptor‐related protein‐1 through GPC4 apparently promotes tau spreading (Saroja et al. 2022). Aβ is a major component of senile plaques and vascular amyloid in AD and appears to promote the expression of agrin and GPC1 in cultured brain pericytes associated with Aβ fibrils attached to the cell membrane, contributing to their accumulation in AD lesions (Timmer et al. 2009; Watanabe et al. 2004).

### 7.4 Agrin Roles in Neurodegenerative Diseases

Agrin (Smith and Hilgenberg 2002), was originally proposed as a stabilizing synaptic organizer (Kröger and Schröder 2002; Nastuk and Fallon 1993; Ngo et al. 2007) but further studies have now outlined alternative roles for agrin in neural tissues (Bezakova and Ruegg 2003; Daniels 2012). Agrin associates with α‐synuclein in Lewy bodies in PD, accelerating both the formation of insoluble α‐synuclein protofibrils and fibril formation (Liu et al. 2005). Aβ modulates the expression of agrin and GPC1, contributing to their accumulation in AD lesions (Timmer et al. 2009). Agrin also binds to β‐A peptide and is localized to amyloid deposits in AD (Cotman et al. 2000; van Horssen et al. 2002). Agrin also accumulates in regions of vascular damage in the brain (Berzin et al. 2000). Agrin abnormalities observed in AD are closely linked to β‐amyloid deposition (van Horssen et al. 2002; van Horssen et al. 2001), with altered agrin expression in the microvasculature and brain parenchyma contributing to AD pathogenesis (Donahue et al. 1999).

### 7.5 Serglycin Has Roles in Neurodegenerative Disorders

Serglycin is upregulated in activated microglia following stroke, where it exacerbates brain injury and inflammation. SRGN promotes tumorigenesis through regulation of NF‐κB, AKT, MAPK/ERK, and β‐catenin signaling and is a biomarker of aggressive cancers such as glioblastoma multiforme (GBM) (Manou, Bouris, et al. 2020; Manou, Karamanos, and Theocharis 2020). Elevated SRGN expression correlates with increased TGFβRI and CXCR‐2 levels and poor patient survival.

Fibroblasts exposed to conditioned media from SRGN‐positive GBM cells show enhanced proliferation, migration, and elevated expression of CXCL‐1, IL‐8, IL‐6, IL‐1β, CCL‐20, CCL‐2, and MMP‐9. This activation depends on SRGN expression and CXCR‐2 signaling in both fibroblasts and GBM cells (Manou et al. 2024). High SRGN expression in gliomas strongly predicts poor prognosis and correlates with mast cell infiltration (Põlajeva et al. 2011; Roy et al. 2017). Glioma cells express the mast cell chemoattractant CXCL12, while mast cells express its receptor CXCR4. Upon degranulation, SRGN‐containing granules release active proteases and cytokines that promote tumor progression.

### 7.6 The ApoA ε4 Allele Predisposes to Neurodegeneration

The ApoA ε4 allele is associated with increased risk of development of cerebral amyloid angiopathy and age‐related cognitive decline. By better understanding these aggregative processes it may be possible to therapeutically target Tau and protein deposition (Fenili and McLaurin 2005; Holmes and Diamond 2014; Moussaud et al. 2014; Raulin et al. 2022; Yamazaki et al. 2019) by designing compounds that block transcellular propagation of amyloid, Tau, and α‐synuclein protein deposition and cellular disruptions that lead to neurodegenerative pathologies in AD, PD, ALS, and HD. Perlecan also has roles in pathological protein deposition in neurodegenerative conditions such as AD (Castillo et al. 1997; Maresh et al. 1996; Rosenmann et al. 2004; Snow et al. 2021). Perlecan domain II LDLR (Murdoch et al. 1992; Noonan et al. 1991) ApoE interactions may potentiate pathological protein deposition in neurodegenerative processes. Perlecan regulates cell signaling and membrane trafficking associated with neurodegeneration including uptake and export of Tau, deposition of amyloid precursor protein‐derived peptides, and regulation of autophagy. Disruption of cellular processes in AD through alterations in HS structure are expected to contribute to these changes. HS could thus be targeted using HS biomimetics to counter these AD‐associated pathological processes countering neurodegenerative pathology (Schultheis et al. 2023; Zhang et al. 2014).

### 7.7 Enigmatic 3‐*O* HS Sulfation in Normal Tissue and in Neurodegerative Conditions

Sulfation at the 3‐*O* position in HS chains is rare (Thacker et al. 2014) but is of functional importance in interactions with AT in the control of coagulation. Interaction of the O‐3 HS sulfation motif with HCII enhances the inhibition of thrombin. O‐3 HS sulfation motifs promote Wnt, Shh, and FGF‐FGFR interactions operative in tissue morphogenesis. Viral infection with SARS CoV‐2 in COVID 19 disease is enhanced by interaction of the spike protein with O‐3 HS. O‐3 HS sulfation motif interactions with Tau enhance pathological protein aggregate formation in neurodegenerative diseases. The O‐3 sulfation motif in HS is also interactive with NRP‐1 and NRP2 transmembrane receptors that are mediators of neuronal guidance and angiogenesis. NRPs bind members of the Class 3 semaphorin family to regulate neuronal guidance, and also interact with the VEGF angiogenic family promoting cerebrovascular development in the CNS/PNS. HS displays the most diverse “sulfation code” of any GAG (Holmes et al. 2022), enabling it to selectively recognize and interact with many proteins (Chittum et al. 2021; Jin et al. 1997; Zhao et al. 2020). This is evidenced in the interactive proteins documented in HS interactome databases (Ori et al. 2011; Turnbull 2010; Vallet et al. 2022). Seven isoforms of 3‐*O*‐sulfotransferases (HS3ST‐1, ‐2, ‐3_A_, ‐3_B_, 4, ‐5, and ‐6) introduce 3‐*O* sulfation motifs into HS chains (Thacker et al. 2014). Proteins that preferentially bind to the 3‐*O*‐sulfation motif in HS include AT (Raghuraman et al. 2006), FGFR (McKeehan et al. 1999), glycoprotein D (Liu et al. 2002), cyclophilin B (Deligny et al. 2010), neuropilin‐1 (Thacker et al. 2016), HCII (Sankarayanarayanan et al. 2017), tau glycoprotein (Zhao et al. 2020), and SARS CoV‐2 spike glycoprotein (Hayashida et al. 2022). HS sulfation sequences flanking the 3‐*O* sulfation region also provide specificity to these interactive 3‐*O* HS sequences.

#### 7.7.1 Promotion of Pathological Protein Aggregation by O‐3 HS Sulfation

The binding of Tau, β‐amyloid, and α‐synuclein to the neuronal cell surface can promote their cellular uptake and seeding of protein aggregates via the brain micro‐vasculature. The GAG‐binding requirements of recombinant tau, α‐synuclein, and β‐amyloid fibrils with HS have been determined to determine the critical size and sulfation positions required for aggregate formation (Stopschinski et al. 2018). Biochemical, cell‐based assays and CRISPR/Cas9 gene knockout studies to specific HS biosynthetic and HS modification enzymes including EXT1, EXT2, EXTL3, NDST1, and HS6ST2 have all been examined. Tau aggregate formation requires 6‐O sulfation in HS, whereas α‐synuclein and β‐amyloid have less stringent HS binding requirements. Knockout of EXT1, EXT2, EXTL3, or NDST1, but not HS6ST2, reduces α‐synuclein uptake, but its requirements for protein deposition are less stringent. Carbohydrate microarrays are extremely useful rapid methods for the determination of interactions between proteins and HS species and show tau efficiently binds to heparin and 2‐O‐desulfated heparin; however, 6‐O‐desulfation, N‐desulfation, or O‐desulfation abolished this binding. Tau is one of a few proteins which preferentially binds to 3‐O‐sulfated HS (Chiti and Dobson 2006); α‐synuclein and β‐amyloid fibrils each require O‐sulfation for binding. Amyloid fibrils (Li and Liu 2020; Makin et al. 2005) have pivotal roles in the pathogenesis of neurodegenerative diseases (Chiti and Dobson 2006). Aggregates of prion protein (Colby and Prusiner 2011) or secretase‐generated protein fragments produce highly ordered toxic amyloid oligomers, small aggregates, and amyloid fibrils (Hoppener et al. 2000). The extracellular accumulation of amyloid beta proteins in neuritic plaques and fibrillary tangles are hallmarks of AD (Ariga et al. 2010).

### 7.8 Concluding Remarks

A summary of the major attributes of HSPGs

1. Central regulation of cell signaling. HSPGs facilitate cell signaling, serve as co‐receptors, and bind growth factors like FGF‐2 to precisely regulate cellular activity and gene expression.
2. Essential for tissue homeostasis, development, and repair. HSPGs play critical roles in tissue homeostasis, fine‐tune tissue development, and ECM remodeling.
3. Key roles in CNS and PNS function. HSPGs support neurogenesis, axonal guidance, synaptogenesis, synaptic function, and neuronal repair, highlighting their importance in maintaining nervous system function in health and disease.
4. Support stem cell niches and neuroprogenitor development. Specific HSPGs such as perlecan are essential components of neural stem cell niches, regulating development of stem cell lineages with roles in the growth of defined brain regions.
5. Highly versatile molecular interactions. HS chains interact with a wide range of cytokines, growth factors, receptors, morphogens, and matrix proteins, underpinning their diverse regulatory roles in physiological processes.
6. Evolutionary molecular recognition and information rich cell instructional capability. The conservation and structural diversity of HS highlight its importance in survival and its role in the “glyco‐code”, a complex system of cell–cell communication that rivals DNA/RNA and protein systems in its complexity.

Negative aspects of HSPGs

1. Some alterations in HSPG structure can induce tissue dysfunction in disease. Changes in HS synthesis/sulfation patterns are linked to conditions such as cancer, diabetes, atherosclerosis, and neurological disorders.
2. Template‐independent synthesis increases complexity. HS structures are not directly encoded by the genome but generated by enzymatic networks, leading to extreme structural diversity complicating analysis, modeling, and therapy.
3. Challenging to model experimentally. HS complexity makes it challenging to understand protein‐HS interactivity in health and disease. Recent significant advances in glycomics, however, are aiding in such determinations and GAG isomer distributions can now be determined in complex diseased tissues.

### 7.9 Recent Developments in HSPG Research

Although glycans make up less than 1% of the mass of brain tissues, N‐glycans, O‐glycans, and gangliosides have profound effects on brain physiology and disease. Advances in glycoscience position brain glycosylation as a promising source of diagnostic biomarkers and therapeutic targets for complex neurological disorders (Hanamatsu et al. 2025; Kurogochi et al. 2025; Mernie and Zaia 2026; Onigbinde et al. 2025; Palomino et al. 2026; Seo et al. 2025; Zulu et al. 2018). It is now possible to map the distribution of CS isomer distributions in normal and neurodegenerative tissues such as ischemic stroke; this methodology indicates that the complexities of HSPGs and their varied GAG isomer distributions may also be examined in a similar manner (Palomino et al. 2026).

Significant advances in glycomics analytical methodology have greatly aided analysis of the complexity of HS. High throughput automated methodology (Shubhakar et al. 2015) and the use of AI to analyze glycosylation data (Li et al. 2022) are significant advances that enable comprehensive glycomic characterization of the glycocalyx of cells (Li et al. 2020). Bioinformatics (Fu et al. 2025) permits quantitative analysis of large glycomics datasets (Lu et al. 2025; Pučić‐Baković et al. 2026). Mass spectrometric methods have improved the elucidation of the HS glyco‐code (Grabarics et al. 2022; Wu et al. 2026) and quantification of HS‐protein interactions (Bui et al. 2026), spatial molecular imaging (Ribas et al. 2025), spatial glycomics, and glycoproteomics (Boottanun et al. 2025). Advanced anion exchange chromatography coupled with mass spectrometry has improved the elucidation of HS structure (Yi et al. 2025). The GAGOme database is a cell‐based library of displayed glycosaminoglycan features (Chen et al. 2018). Glycosyltransferases have proven useful in the examination of the biology of glycans (Kofsky et al. 2023). These glycomic methodologies provide high sensitivity analysis of glycans facilitating their potential application in biomedicine (Lageveen‐Kammeijer et al. 2022), and of biomarker discovery in human diseases (Onigbinde et al. 2025).

## 8 Conclusions

This review highlights the central roles of HSPGs in brain development and synaptic function, including axonal guidance, synapse formation, and cell type–specific neural connectivity. Distinct HSPG expression profiles help shape precise neural circuits. HSPG dysregulation is associated with impaired neurodevelopment, cognitive decline, and pathological changes in brain tissues emphasizing the importance of HSPGs in both normal nervous system function and disease‐associated tissue dysfunction. The evolutionary conservation and structural diversity of HS underscore its importance within the glyco‐code, an information‐rich system of cell–cell and cell‐ECM communication that rivals DNA/RNA and protein systems in complexity. This structural complexity has historically made HSPG analysis challenging; however, recent advances in glycomics and molecular imaging are improving analytical resolution and enabling the assembly of tissue atlases of GAG isomer distributions in healthy and diseased neural tissues. Mapping HS‐interacting proteins in these contexts will provide invaluable insight into disease mechanisms and may identify future therapeutic targets for complex neurological conditions.

## Author Contributions

The author takes full responsibilities for this article.

## Funding

This work was supported by Melrose Personal Research Fund.

## Conflicts of Interest

The author declares no conflicts of interest.

## Supporting information


**Data S1:** Transparent Science Questionnaire for Authors.

## Data Availability

This is a narrative review no new data was generated.
